# Explainable deep learning for early sepsis detection from ICU time-series data using XAI techniques

**DOI:** 10.1038/s41598-026-61652-x

**Published:** 2026-07-27

**Authors:** Anas Mahmoud, Hamza Abdelmoreed, Hossam Amir, Mohamed Ehab, Rana Abdelfattah, Mayada HadHoud

**Affiliations:** 1 School of Computational Sciences and Artificial Intelligence (CSAI), Zewail City of Science and Technology, Giza, Egypt; 2 Department of Computer Engineering, Faculty of Engineering, Cairo University, Cairo, Egypt

**Keywords:** Computational biology and bioinformatics, Diseases, Health care, Mathematics and computing, Medical research

## Abstract

Sepsis is one of the most deadly illnesses with a high risk of mortality. Consequently, identifying it at the beginning of illness symptoms is crucial and plays a key role in improving patient outcomes. This study presents a customized solution for the early detection of sepsis with an emphasis on the use of interpretability and explainability techniques, utilizing a range of machine learning approaches and interpretable artificial intelligence methods. The database on which this research study is based has many problems; the main ones being large data gaps and class disparities. Employing robust methods, precise categorizations, and rigorous computations, Approximately 12 diverse models were developed and optimized. With ROC-AUC indicators of 0.9566 and 0.9595 and F1 scores of 0.85 and 0.85 respectively, Bidirectional Long Short-Term Memory (BiLSTM) and Temporal Convolutional Network (TCN) models performed better than conventional models in terms of sepsis prediction. These two approaches have shown remarkable progress in detecting clinical patterns while avoiding false negative results—an essential aspect of the medical field. To assess model performance and offer clear insights into model predictions, interpretation-based techniques were employed. This improved clinical confidence and facilitated well-informed decisions in crucial medical diagnoses.

## Introduction

Sepsis is a significant global health concern, causing approximately 11 million deaths annually and accounting for about 20% of all global mortality^1^. When a body encounters an infection, the immune system releases cytokines and other inflammatory mediators. While this response aims to eliminate pathogens, excessive or uncontrolled activation can cause tissue damage, multiple organ failure, and even death^1^. There is no single definitive test to diagnose sepsis^2^. Clinicians depend on a combination of clinical signs, laboratory tests, and biomarkers to evaluate whether a patient is septic or non-septic^3^. Early detection of these symptoms enables preventative measures to reduce mortality^4^.

Despite the urgent need for early detection, current diagnostic approaches are restricted by complexity and variability inherent in sepsis progression^5^. Traditional methods may not capture changing patterns in patient data that can signal sepsis onset. As highlighted in^2^, sepsis presents a diagnostic challenge because it is not directly recognizable. These challenges are compounded by substantial data quality and reliability issues. For instance, a widely used dataset is the PhysioNet 2019 Challenge dataset, which has more than 90% missing values and demonstrates severe class imbalance^3^.

Many studies have investigated various Machine Learning (ML) and Deep Learning (DL) models for early detection of sepsis, but several gaps persist. A notable limitation in several studies, including those by Lyra et al.^6^, Wu et al.^7^, and Liao et al.^8^, is the absence of model interpretability. Algorithms like Long Short-Term Memory (LSTM), Gated Recurrent Unit (GRU), and Extreme Gradient Boosting (XGBoost) achieved moderate success in sepsis prediction but did not implement interpretable techniques such as Shapley Additive exPlanations (SHAP) or Permutation feature importance (PFI) to clarify their decision-making processes. This lack of transparency limits interpretability and impedes model improvement.

Although some research utilized feature selection techniques, few studies have compared the effects of different selection methods on model performance. Furthermore, management of missing data and class imbalance is inconsistent, with limited assessments of imputation methods or balancing strategies. This problem is especially apparent in the PhysioNet 2019 dataset, where about 90% of records belong to the non-septic class, and over 90% of values are missing for many laboratory variables due to the intermittent nature of lab testing rather than regular hourly sampling^3^.

This study directly addresses the critical need for reliable early detection of sepsis, a task often hindered by highly incomplete clinical data and severe class imbalance. To overcome these challenges and improve the credibility of predictive models in clinical practice, an integrated framework is proposed that combines advanced machine learning with Explainable AI (XAI) techniques.

The key contributions of this research are summarized as follows:

1. Development of a robust data preprocessing pipeline specifically designed to address extreme missingness and class imbalance inherent in sepsis datasets, ensuring the generation of more balanced and informative training data.
2. Enhancement of model interpretability for early sepsis prediction through the application of diverse XAI methods, including SHAP and Permutation Feature Importance, offering comprehensive insights into the decision-making processes of machine learning models.
3. Provision of clinically meaningful explanations that not only identify the most influential features driving predictions but also pinpoint the temporal significance of these features, thereby fostering clinical confidence and enabling more informed decision-making in critical care environments.

The rest of this paper is organized as follows: Section Literature review reviews related work on sepsis prediction and machine learning in healthcare. Section Methodology outlines the methodology, including data preprocessing, model development, and explainability techniques. Section Experimental results & discussion presents and discusses the experimental results. Finally, Section Conclusion & future work summarizes the findings and suggests directions for future research and clinical application.

## Literature review

To contextualize this research, twelve key studies applying machine learning (ML) and deep learning (DL) techniques for early sepsis prediction have been reviewed. These studies were specifically selected based on their relevance to the progression of sepsis prediction methods, their methodological diversity across traditional ML and DL, and their focus on addressing critical challenges such as data imbalance, missingness, and temporal modeling. This curated selection ensures a comprehensive understanding of the current landscape and its limitations.

Traditional ML methods have been extensively utilized for sepsis prediction. Lyra et al.^6^ employed Random Forest (RF) classification to address clinical data imbalance, achieving a mean Area Under the Receiver Operating Characteristic Curve (AUROC) of 0.81. Wu et al.^7^ proposed a customized down-sampling approach combined with a dynamic sliding window, resulting in an AUC of 0.89. Similarly, Nirgudkar and Ding^9^ applied imputation strategies alongside a weak ensembler technique, obtaining an internal validation accuracy of 93.45%. Kong et al.^10^ developed Gradient Boosting Machine (GBM) and RF models, reporting superior performance for GBM at 84.5% accuracy, compared to other models ranging between 77% and 83%. More recently, Vandana and Chhikara^11^ evaluated various ML models with feature selection techniques for ICU sepsis prediction, achieving a peak accuracy of 95% using a Decision Tree model combined with Information Gain.

The adoption of DL methods has expanded significantly, particularly due to their effectiveness in capturing temporal dependencies within clinical data. Lipton et al.^12^ addressed the challenge of missing data by employing Recurrent Neural Networks (RNNs) to impute missing values in clinical time series. Liu et al.^13^ introduced a Heterogeneous Event Aggregation (HEA) framework enhanced with LSTM to model complex temporal interactions within Electronic Health Records (EHRs), achieving an AUC of 0.8224 with their 16-head HEA-LSTM model. Apalak and Kiasaleh^14^ utilized Temporal Convolutional Networks (TCNs) on the MIMIC-III dataset, focusing on Heart Rate Variability (HRV) features, and reported an AUROC of 0.82. Oei et al.^15^ applied a BiLSTM model to the same dataset, reaching an AUROC of 0.85. Liao et al.^8^ conducted a comparative study on the PhysioNet 2019 dataset, benchmarking DL models such as InceptionTime and TCN against traditional ML methods like RF and XGBoost. Their results demonstrated that DL models outperformed traditional approaches, with TCN and InceptionTime achieving AUROC scores of 0.92 and 0.90, respectively.

Despite these advancements, several critical gaps persist within the existing literature. One prominent limitation is the lack of model interpretability; many studies have not incorporated explainability tools such as SHapley Additive exPlanations (SHAP) or Permutation feature importance (PFI), which are essential for building trust in clinical settings. Additionally, there remains an inconsistent treatment of missing data and class imbalance, which hinders model reliability and generalizability. The limited comparative evaluation of feature selection methods and the underutilization of ensemble techniques further restrict the robustness and optimization of predictive models.

## Methodology

### Research gap & proposed approach

A detailed review of existing work highlights three persistent limitations that reduce clinical viability and trustworthiness of current ML-based systems for sepsis prediction.

First, many models lack interpretability, which poses a serious challenge in medical settings. Although models including XGBoost, LSTM, and GRU have achieved strong predictive performance in studies by Lyra et al.^6^, Wu et al.^7^, and Liao et al.^8^, they generally do not integrate XAI tools. As a result, these models function as black boxes, limiting clinicians’ ability to understand or validate their outputs. This lack of transparency undermines clinical trust and makes it difficult to meet regulatory standards for artificial intelligence (AI) in healthcare.

Second, the issue of data availability and quality remains largely unresolved. Specifically, this includes challenges related to missing data, noisy measurements, and incomplete clinical records which are common in real-world healthcare datasets. In the PhysioNet 2019 dataset^3^, over 90% of values for some lab features are missing, and non-septic patients account for an overwhelming majority of cases. Such gaps in data can impair model learning, increase bias, and diminish predictive reliability. Prior studies apply different preprocessing methods for imputation and resampling^12,16^, but very few offer comparative analyses to determine which strategies are most effective. This inconsistency raises concerns about the reproducibility and reliability of the resulting models.

Third, while feature selection is frequently mentioned^11^, most existing work does not rigorously compare multiple selection methods. Without evaluating different strategies side by side, opportunities to improve both model performance and interpretability may be missed. An optimized feature set can simplify models while retaining essential clinical insight.

To tackle the challenges of early sepsis detection and the inherent complexities of clinical data, the proposed framework combines advanced temporal modeling with a comprehensive suite of explainability techniques. Specifically, it incorporates a range of Explainable AI (XAI) methods, including SHAP, PFI, Permutation Feature Importance (PFI), Integrated Gradients, and Accumulated Local Effects (ALE). This approach provides detailed, multi-dimensional insights into the reasoning behind model predictions. This framework, which leverages XAI alongside machine learning, is designed to transform complex neural network decisions into clear, trustworthy insights that clinicians can readily interpret and act upon.

Methodological approach emphasizes evaluation of BiLSTM and TCN models due to their superior capacity for capturing temporal dependencies in sequential clinical data—a critical requirement for early sepsis detection, where temporal evolution of patient parameters contains essential predictive information^14,17^. By combining these advanced temporal modeling capabilities with comprehensive explainability tools, this study aims to bridge the gap between computational sophistication and clinical practicality, ultimately advancing the development of trustworthy AI systems for critical medical diagnostics.

### Data preparation

#### Used dataset

This study employs the PhysioNet 2019 Challenge dataset^3^ as the primary source of multivariate time series data for sepsis prediction. The dataset includes comprehensive clinical records of ICU patients with rich temporal features crucial for sepsis detection. Focusing on the PhysioNet 2019 dataset because it is widely regarded as the state-of-the-art benchmark for sepsis prediction tasks. The dataset is extensively used in the research community due to its richness, diversity of clinical variables, which fosters standardized evaluation across studies. Leveraging such a comprehensive dataset ensures that our findings are comparable to existing work and applicable to real-world clinical scenarios.

**Table 1:** Clinical and laboratory features used from the PhysioNet 2019 dataset.

Feature	Medical description
HR	Heart rate (beats per minute)
O_2_Sat	Peripheral oxygen saturation (%)
Temp	Body temperature (°C)
SBP	Systolic blood pressure (mmHg)
MAP	Mean arterial pressure (mmHg)
DBP	Diastolic blood pressure (mmHg)
Resp	Respiratory rate (breaths per minute)
BaseExcess	Blood base excess indicating metabolic status
FiO_2_	Fraction of inspired oxygen (%)
pH	Blood acidity/alkalinity level
PaCO_2_	Partial pressure of carbon dioxide (mmHg)
BUN	Blood urea nitrogen (renal function marker)
Calcium	Serum calcium level
Creatinine	Serum creatinine (kidney function indicator)
Glucose	Blood glucose level
Magnesium	Serum magnesium level
Potassium	Serum potassium level
Hct	Hematocrit (proportion of red blood cells)
Hgb	Hemoglobin concentration
WBC	White blood cell count (infection indicator)
Platelets	Platelet count (clotting function)
Gender	Patient biological sex
ICULOS	ICU length of stay (hours since ICU admission)
SepsisLabel	Binary label indicating sepsis onset
Patient_ID	Unique patient identifier

The dataset encompasses vital signs, laboratory tests, demographic information, and temporal indicators alongside the binary sepsis outcome, with detailed clinical descriptions provided in Table 1. This comprehensive feature set enables multidimensional assessment of patient physiological status and sepsis progression dynamics.

#### Data preprocessing & handling imbalance

Data preprocessing involved handling missing values using advanced imputation techniques such as k-nearest neighbors (KNN) imputation and forward filling methods. To address the severe class imbalance, SMOTE (Synthetic Minority Over-sampling Technique) and class weighting strategies were employed. These methods ensure a balanced representation of septic and non-septic cases, enhancing the robustness and generalizability of predictive models.

### Predictive models

In this research, several DL and ML models are implemented to identify the most effective approach for early sepsis prediction using multivariate clinical time series data. The selection of models is driven by their ability to capture complex temporal relationships, represent diverse clinical variables, and maintain computational efficiency and robustness against data irregularities. This section provides a detailed overview of the models explored in this study, categorized into traditional ML models and DL time series models.

#### Traditional machine learning models

RF is an ensemble method that aggregates the predictions from a multitude of decision trees trained on bootstrapped samples of the training set^10,18^. Each tree is grown based on a randomly selected subset of features to reduce inter-tree correlation. In this study, the maximum depth was controlled and Gini impurity was employed as the splitting criterion. The model exhibited high interpretability and robustness against overfitting, making it particularly suitable for clinical tabular data. RFE was further applied to enhance feature selection performance^10,18^, with the complete configuration summarized in Table 2.

XGBoost, a gradient boosting framework, was also employed. This method builds models sequentially while applying regularization to mitigate overfitting. In the present implementation, a binary logistic objective was used and hyperparameters were optimized through Bayesian search. Generalization was further supported via early stopping across five-fold cross-validation. The final model demonstrated high predictive performance, particularly in imbalanced settings^17,19,21^. The configuration details are outlined in Table 2.

#### Traditional machine learning models

**Table 2:** Model configurations.

Model	Key parameters
RF	n_est=100, bootstrap, Gini min_split=10, min_leaf=5 max_depth=None
XGBoost	depth=7, lr=0.124 subsample=0.97, colsample=0.84 gamma=0.50, min_child=8 alpha=5.74, lambda=2.45 n_est=1000
Logistic Reg.	L-BFGS, max_iter=1000 Standard scaling, 80–20 split

In addition, RF was utilized with 100 estimators, optimized splitting thresholds, and restricted tree depths to minimize variance. The model used characteristic importance scores to identify clinically influential predictors of sepsis^6^. The set of engineered features used in these models is detailed in Table 3.

**Table 3:** Engineered features.

Feature	Formula
Pulse pressure	SBP-DBP
Cardiac output	(SBP-DBP) × HR
Shock index	HR/SBP
Modified SI	HR/MAP
Z-scores	SBP, DBP, HR, MAP

#### Deep learning time series models

Several DL architectures were evaluated to capture the temporal dynamics of multivariate clinical time series data. These models are grouped into recurrent-based and convolution-based frameworks.

*Recurrent Models* incorporate hidden states and temporal feedback mechanisms. A baseline RNN with 64 hidden units, global average pooling, and a sigmoid output layer was developed, employing dropout regularization to reduce overfitting^12^. LSTM networks were constructed by stacking LSTM layers with dropout and global average pooling, optimized using the Adam optimizer^12^. BiLSTM was then introduced to process sequences in both forward and backward directions, thereby enhancing context modeling. This architecture included stacked BiLSTM layers, batch normalization, and dropout regularization^22^. Finally, GRU networks were employed for their parameter efficiency, utilizing simplified gating structures. GRUs incorporated L2 regularization, dropout, and early stopping strategies^10,18^. The architectural details and regularization strategies of these recurrent models are provided in Tables 4 and 5, respectively.

**Table 4:** Recurrent models: architecture.

Model	Architecture description
RNN	64 hidden units, global average pooling, dense sigmoid output
LSTM	Stacked LSTM layers with dropout, global average pooling
BiLSTM	Two BiLSTM layers, batch normalization, dropout, global average pooling
GRU	GRU layers with simplified gating, global average pooling

**Table 5:** Recurrent models: regularization techniques.

Model	Regularization and optimization
RNN	Dropout regularization to prevent overfitting
LSTM	Dropout, Adam optimizer
BiLSTM	Dropout, Batch normalization
GRU	L2 regularization, Dropout, Early stopping

*Convolutional Models* employ convolutional operations to extract local and hierarchical temporal patterns. TCNs utilize dilated causal convolutions with expanding receptive fields and global max pooling to capture long-range dependencies efficiently^7,14^. The architectural details of these convolutional models are summarized in Table 6.

InceptionTime networks apply multiple convolution kernels of different sizes in parallel (inception modules), combined with dropout and global average pooling for regularization^8,17^. 1D-CNNs use conventional convolution and pooling layers to extract local features, offering computational efficiency albeit with limited long-range modeling capacity^7,9^. Finally, ResNet-1D incorporates residual skip connections into 1D convolutional layers to facilitate deeper temporal representations and improved gradient flow^15,22^. Table 6 summarizes the architectural details of these convolutional models.

**Table 6:** Convolutional models: detailed architecture.

Model	Architecture details
TCN	Conv1D Layers: 3 layers with filters [128, 256, 512], dilation rates [1, 2, 4], ReLU activation
	Regularization: Dropout (0.3) after each Conv1D layer
	Output: Dense layer (1 unit, sigmoid activation)
	Optimizer: Adam, Loss: Binary cross-entropy
InceptionTime	Inception Module: 3 parallel Conv1D paths with kernel sizes [10, 20, 40]
	Regularization: Dropout layer after each parallel path
	Output: Dense layer (1 unit, sigmoid activation)
	Optimizer: Adam, Loss: Binary cross-entropy
1D-CNN	Conv1D Layers: 2 layers - Layer 1: 64 filters (kernel=3), Layer 2: 128 filters (kernel=3), ReLU activation
	Dense Layers: Hidden layer (128 units, ReLU), Output layer (1 unit, sigmoid)
	Optimizer: Adam, Loss: Binary cross-entropy
ResNet-1D	Residual Blocks: 2 blocks - Block 1: 64 filters, Block 2: 128 filters, ReLU activation
	Regularization: Batch normalization within blocks, Dropout (0.3)
	Output: Dense layer (1 unit, sigmoid activation)
	Optimizer: Adam, Loss: Binary cross-entropy

### Data overview and preprocessing

**Fig. 1:**
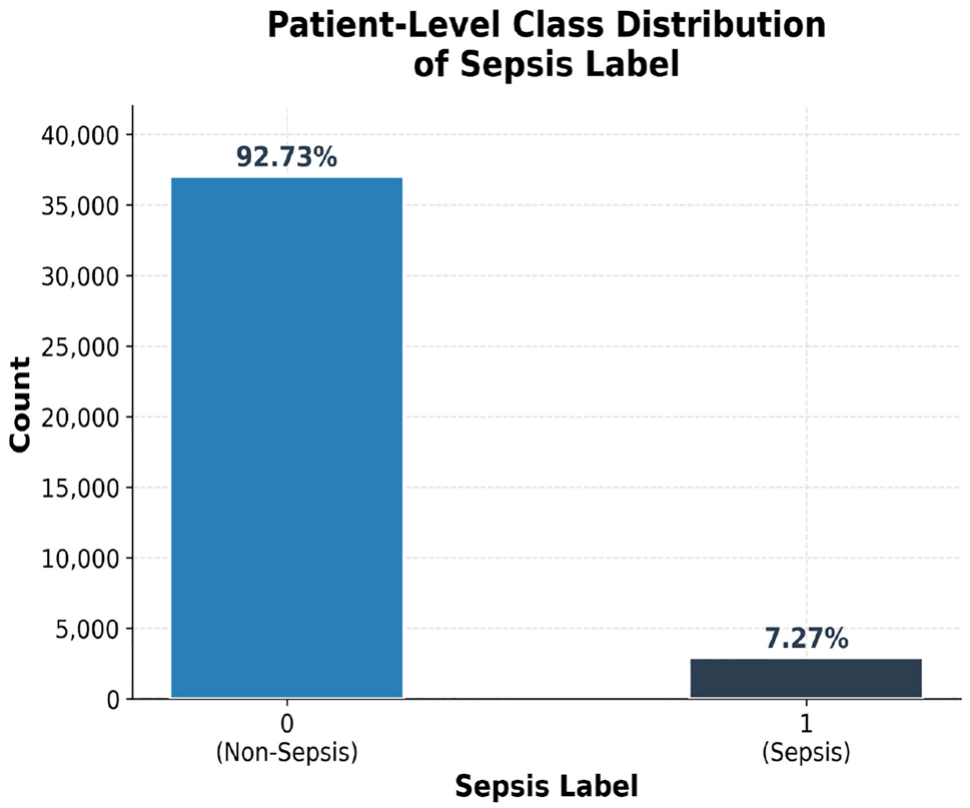
Distribution of classes per patient.

The training dataset consisted of 1.5 million ICU observations from over 40,000 patients ^3^. The class distribution per patient, as shown in Fig. 1, highlights the proportion of septic and non-septic cases.

**Fig. 2:**
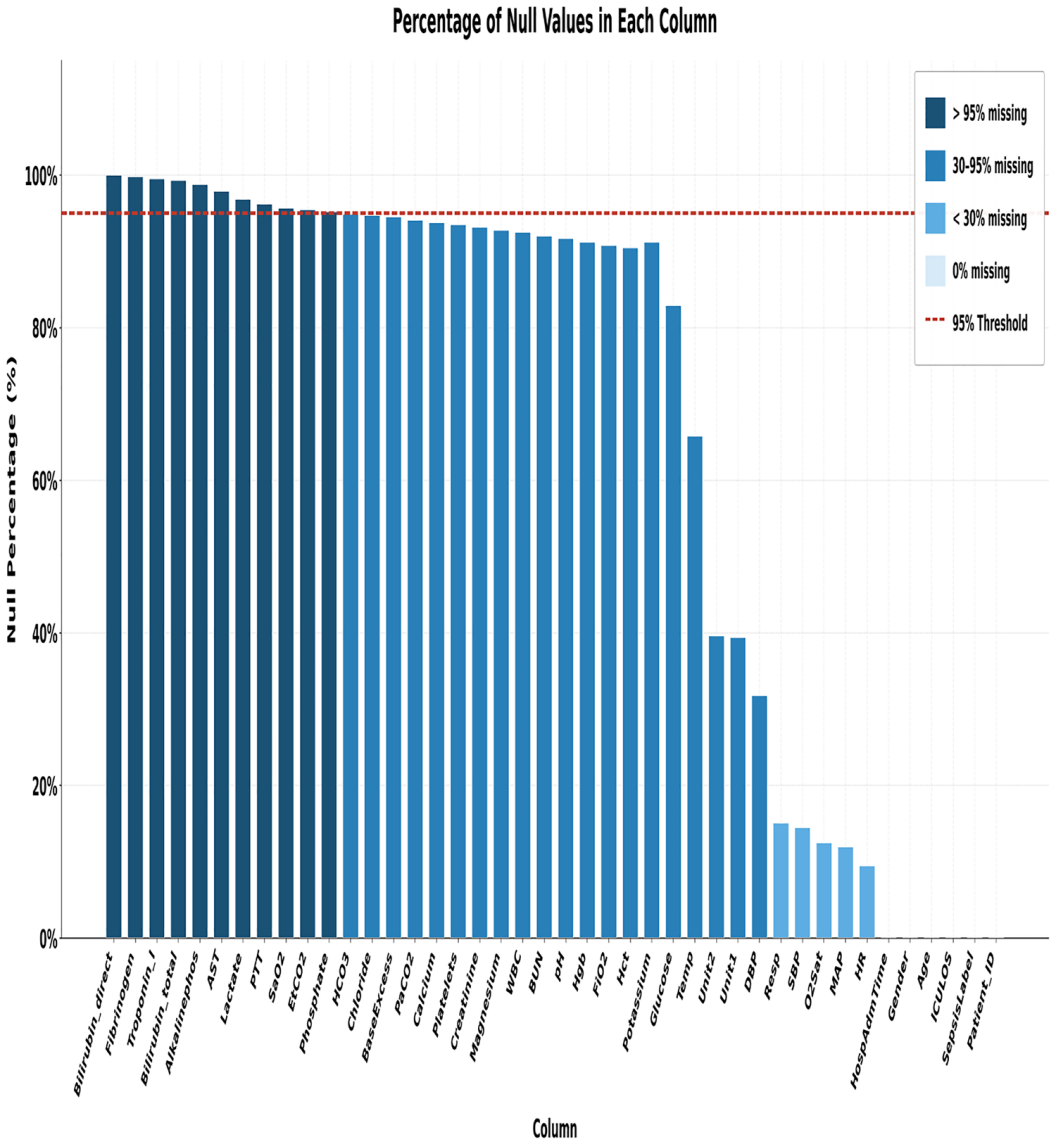
Distribution of null percentage per column.

After applying exploratory data analysis (EDA), the following key findings were observed:

- *Severe Class Imbalance* Only $\sim$7% of patients were labeled as septic (SepsisLabel = 1), with the majority being non-septic. This imbalance is clearly visualized in Fig. 1.
- *Patient-Level Distribution*^3^:
  - Sepsis patients: 2,805
  - Non-sepsis patients: 37,087
- *High Percentage of Nulls Across Most Features* About 25 features have more than 89% nulls. Over 10 lab-related features had >95% missingness (e.g., Bilirubin_direct, Fibrinogen, TroponinI). This high level of missingness is depicted in Fig. 2, which shows the distribution of null percentages across the dataset’s features ^3,12^.

A major issue is that each patient does not have a fixed length of stay, as it ranges from 8 to 336 hours, with an average of 38.48 hours after filtering. This variability is problematic because it prevents trained models from having dynamic input length, which is a key consideration in ICU patient monitoring. While no specific figure illustrates this variability, it is a critical factor affecting the dataset’s structure as analyzed alongside Figs. 1 and 2^3,22^.

#### Handling missing data

**Fig. 3:**
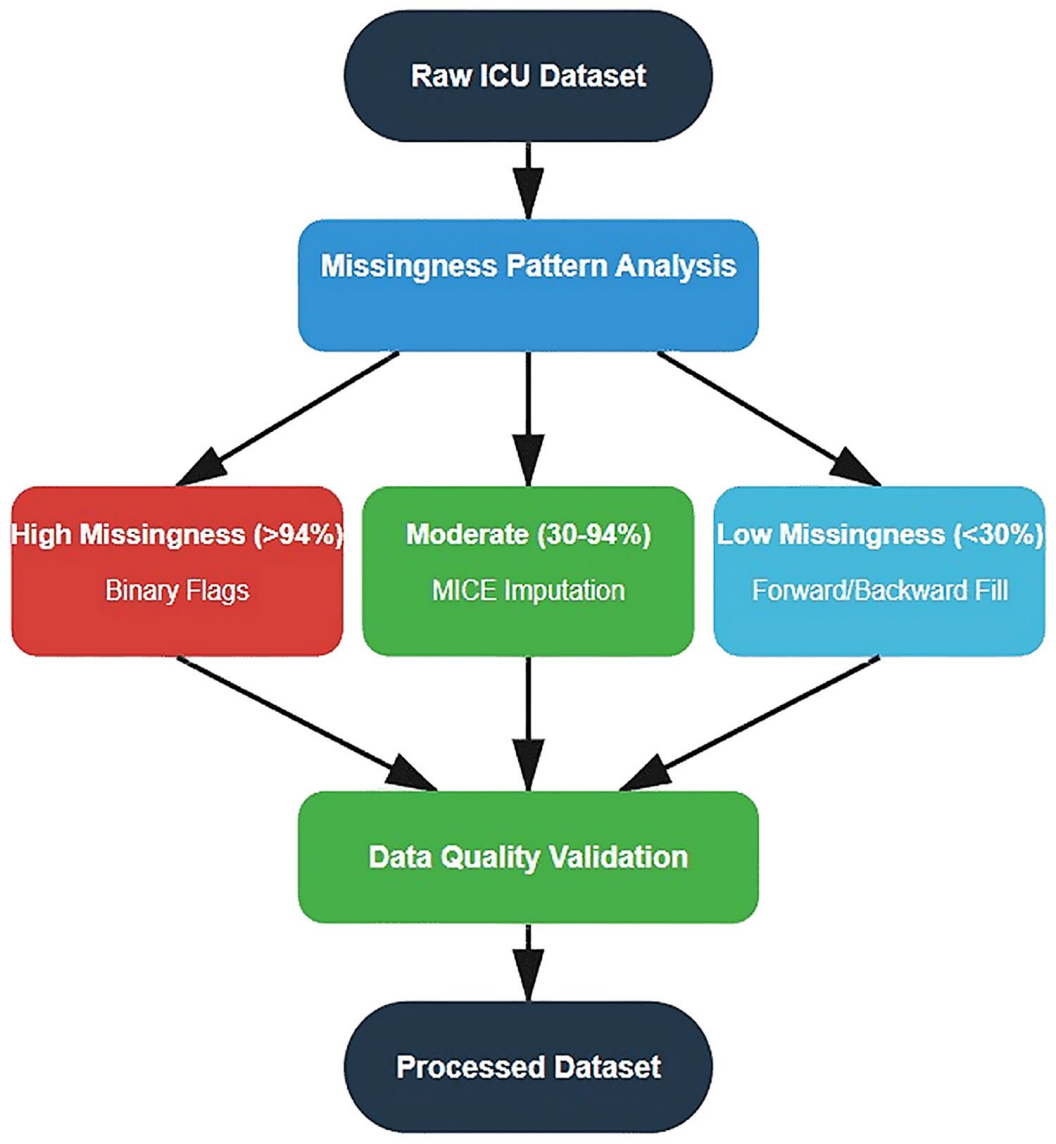
Nulls handling pipeline.

Figure 3 illustrates the complete pipeline for handling missing data in the dataset, where features are systematically divided into three distinct groups based on their missingness percentage. Each group is then subjected to a tailored imputation or handling strategy, justified by the nature of the data and clinical considerations.

These groups are:

1. *Features with > 95% Missingness: Informative Missingness and Binary Flags* This category includes features such as Bilirubin_direct, Fibrinogen, and Lactate, which exhibit a very high percentage of missing values. Instead of outright exclusion, these features were handled by dropping their original values from direct use in modeling but retaining their implicit information. Specifically, binary flags were introduced to indicate whether the corresponding test was performed or not. This approach is rooted in the concept of ”informative missingness,” which posits that the absence or presence of certain clinical measurements can itself be a valuable piece of information for predictive models^12,23^. In an ICU setting, high missingness in lab values often does not signify data corruption but rather reflects deliberate clinical decision-making. Doctors typically order these specific lab tests only when there are particular clinical concerns or symptoms, making their infrequent appearance highly significant^12^. For instance, a bilirubin test might only be ordered if liver dysfunction is suspected, and its presence (or absence) provides diagnostic insight. This allows models to learn from the context of why a test was (or was not) performed, rather than solely from its measured value.
2. *Features with 35–94% Missingness: Multiple Imputation by Chained Equations (MICE)* Features falling within this intermediate range of missingness, such as Glucose, Blood Urea Nitrogen (BUN), and White Blood Cell (WBC), were retained for imputation. These lab tests are generally not measured as frequently as vital signs, and their values are assumed to have less time-sensitive variability, making them suitable for more sophisticated imputation techniques^22^. The Multiple Imputation by Chained Equations (MICE) algorithm^16^ was employed for this purpose. MICE works by building a separate predictive model for each feature with missing values, using all other features in the dataset as predictors. This iterative process generates multiple complete datasets, leading to more robust and realistic imputations compared to single imputation methods.To account for clinical data uncertainty and prevent overfitting, Gaussian noise was added during the imputation process. A specific challenge arose with Diastolic Blood Pressure (DBP), which remained entirely missing for 7,411 patients even after MICE imputation. This scenario typically occurs when MICE cannot impute values due to complete missingness within a patient’s record. To address this, a physiological imputation strategy was implemented: forward and backward filling per patient. Furthermore, if DBP was still missing but Systolic Blood Pressure (SBP) and Mean Arterial Pressure (MAP) were available, the following clinically derived formula was applied to estimate DBP^24^:
  1 $$\begin{aligned} \text {DBP} = \frac{3 \cdot \text {MAP} - \text {SBP}}{2} \end{aligned}$$
3. *Features with < 35% Missingness: Forward and Backward Filling* This group primarily comprises vital signs, such as Heart Rate (HR) and Oxygen Saturation (O_2_Sat), which are frequently and regularly measured in the ICU. Given the clinical assumption that vital signs do not fluctuate significantly over short time intervals^25^, missing values in this category were imputed using a straightforward forward and backward filling approach per patient. This method effectively propagates the last known valid observation forward and the next known valid observation backward to fill gaps, reflecting the relative stability of these measurements within short periods.

To validate that our missing data handling methodology preserves the original data distributions, we computed the Kullback-Leibler (KL) divergence between the original and post-imputation feature distributions Table 7. The KL divergence values demonstrate that our approach maintains data integrity across all features, with particularly low divergence for vital signs (0.004 to 0.042), confirming our strategies preserve the underlying statistical properties. Notably, Calcium exhibits the highest divergence (0.546627) due to substantial outliers and approximately 95% missingness. This validation ensures our imputation methodology does not distort original data characteristics, supporting subsequent modeling reliability efforts.

**Table 7:** KL divergence between scaled original and handled feature distributions.

Feature name	KL divergence
HR	0.022266
O_2_Sat	0.042458
Temp	0.017659
SBP	0.008961
MAP	0.009847
DBP	0.004400
Resp	0.011093
BaseExcess	0.139073
FiO_2_	0.004877
pH	0.172227
PaCO_2_	0.240938
BUN	0.187122
Calcium	0.546627
Creatinine	0.100096
Glucose	0.249988
Magnesium	0.184255
Potassium	0.210672
Hct	0.155876
Hgb	0.112742
WBC	0.034854
Platelets	0.297486

#### Data balancing strategy

**Fig. 4:**
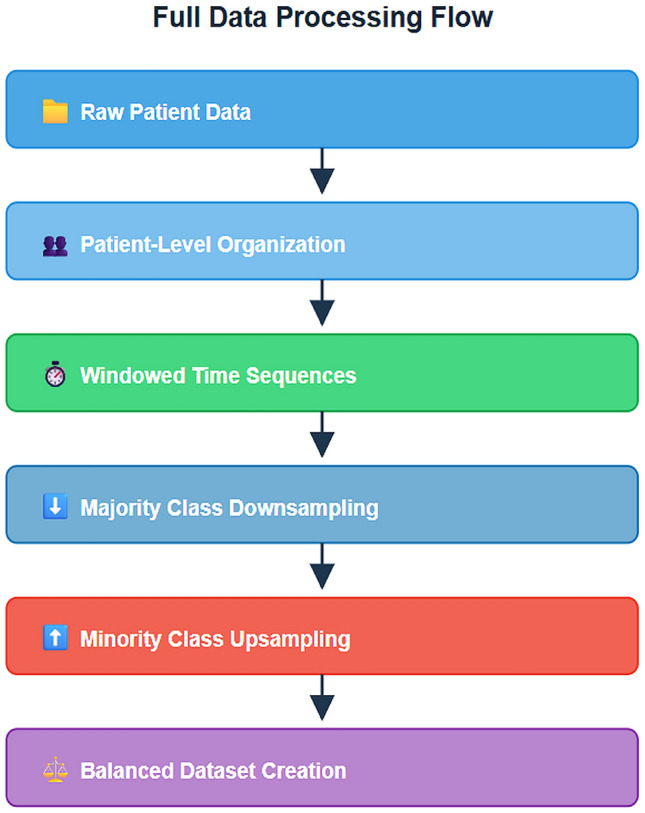
Pipeline for balancing class distribution in patient data.

The dataset was initially organized at the patient level by combining hourly clinical values for each person to efficiently model sepsis predictions, as illustrated in Fig. 4. This organization is crucial for capturing the temporal dynamics associated with sepsis.

The decision tree is used exclusively as a feature importance estimation mechanism and not as a predictive model in the final classification pipeline. Because decision trees are inherently interpretable and can quantify feature relevance through impurity reduction metrics, they provide a computationally efficient and transparent method to identify the most informative physiological variables across temporal sequences. To enable this analysis, each temporal window is flattened into a feature vector while preserving feature identity, allowing the decision tree to estimate global feature salience across the training dataset. This feature importance estimation guides the synthetic sample generation process by restricting perturbations to clinically relevant dimensions rather than applying uniform perturbations across all features. This reduces the risk of introducing unrealistic physiological patterns and improves the biological plausibility of synthetic samples. By prioritizing important features, the augmentation process preserves meaningful physiological structure while increasing representation of minority class patterns. To prevent data leakage and ensure valid evaluation, strict patient level separation was enforced prior to any preprocessing, augmentation, or feature analysis. Specifically, patient records were first partitioned into training, validation, and test sets at the patient level, ensuring that no patient appears in more than one split.

**Fig. 5:**
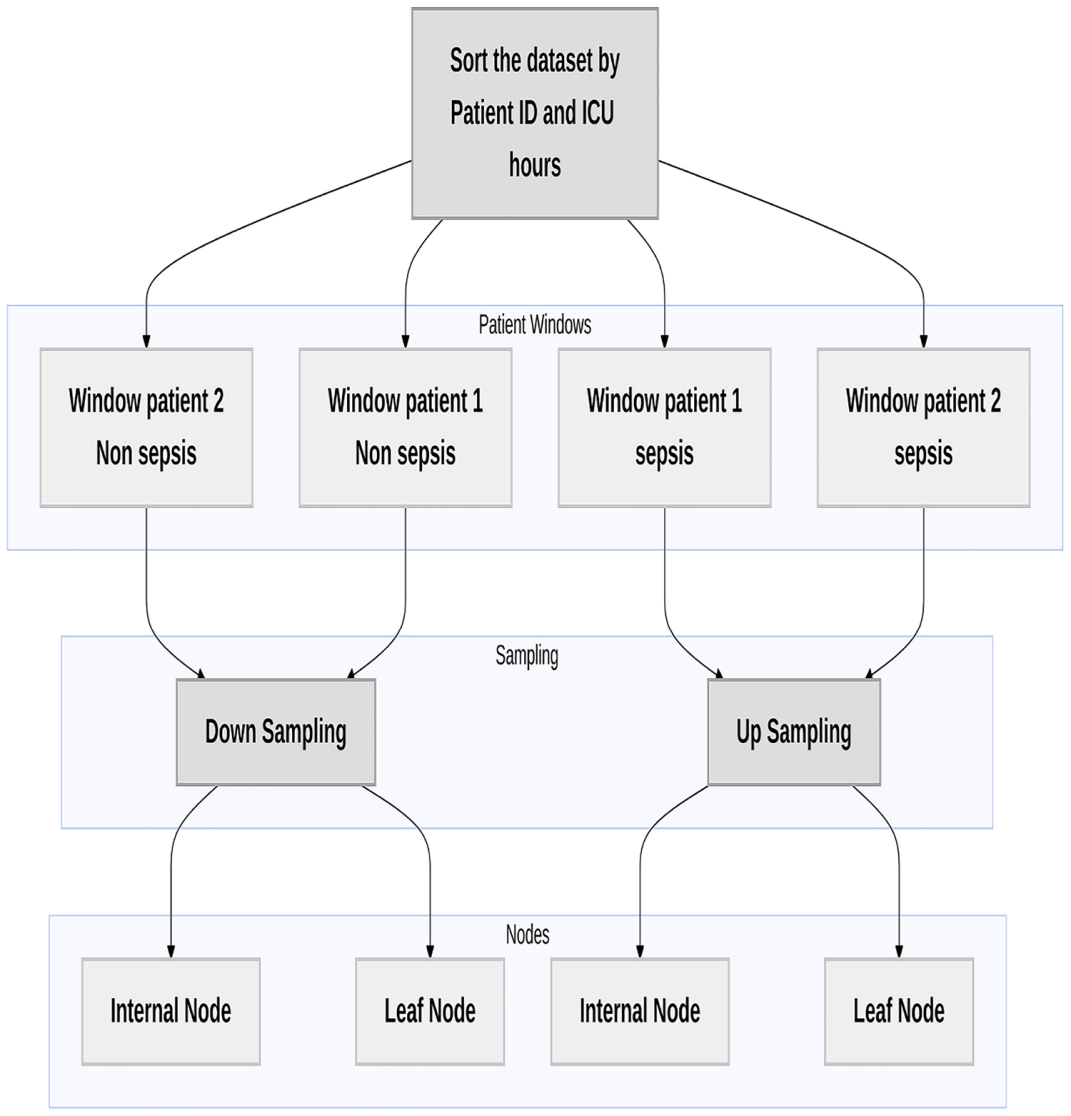
Window-based sampling of patient data to capture temporal dynamics.

To address the challenge of class imbalance while preserving essential temporal information, a window-based sampling approach was implemented, as shown in Fig. 5. Each patient’s timeline was divided into fixed-length overlapping intervals. If any time step within a window indicated a positive sepsis diagnosis, that entire window was classified as sepsis-positive. This method ensures consistency across patients, regardless of their hospital stay duration.

Due to the inherent class imbalance in the dataset—where non-sepsis instances significantly outnumber sepsis cases—a customized two-stage sampling framework was implemented. This strategy ensures that the classifier receives a balanced and informative representation of both classes during training. The full pipeline is outlined in Fig. 5 and comprises the following steps:

1. *Decision Tree-Based Undersampling* To mitigate the dominance of the majority class (non-sepsis), a targeted undersampling strategy was employed. Initially, a random subset (maximum of 50,000) of majority-class samples was flattened and labeled across all time steps. A decision tree classifier (maximum depth of 5) was then trained to learn key discriminative features. Using this model, a representative sample of 35, 000 sequences was drawn, preserving feature diversity and temporal context. This intelligent reduction decreased training time while maintaining clinical relevance.
2. *Decision Tree-Guided Synthetic Oversampling* To increase representation of the minority class (sepsis), biologically-informed synthetic sequences were generated. Specifically, the previously trained decision tree’s feature importance scores guided the perturbation process: synthetic samples were created by injecting Gaussian noise into time-aligned features, with noise magnitudes weighted by their relative importance (only features with importance $> 0.01$ were modified). This process expanded the sepsis class to 27, 000 sequences, enhancing the model’s sensitivity to rare but critical patterns indicative of sepsis onset.
3. *Stratified Train-Test Splitting* The resulting balanced dataset was partitioned into training and testing subsets. A total of 28, 000 non-sepsis and 21, 600 sepsis sequences were allocated to the training set, while the remaining samples were reserved for evaluation. This stratified split ensures unbiased performance assessment and preserves the effects of the prior balancing steps.

This hybrid sampling strategy–combining intelligent data reduction and biologically-aware augmentation—was essential for training a robust and generalizable model under realistic clinical data constraints.

**Fig. 6:**
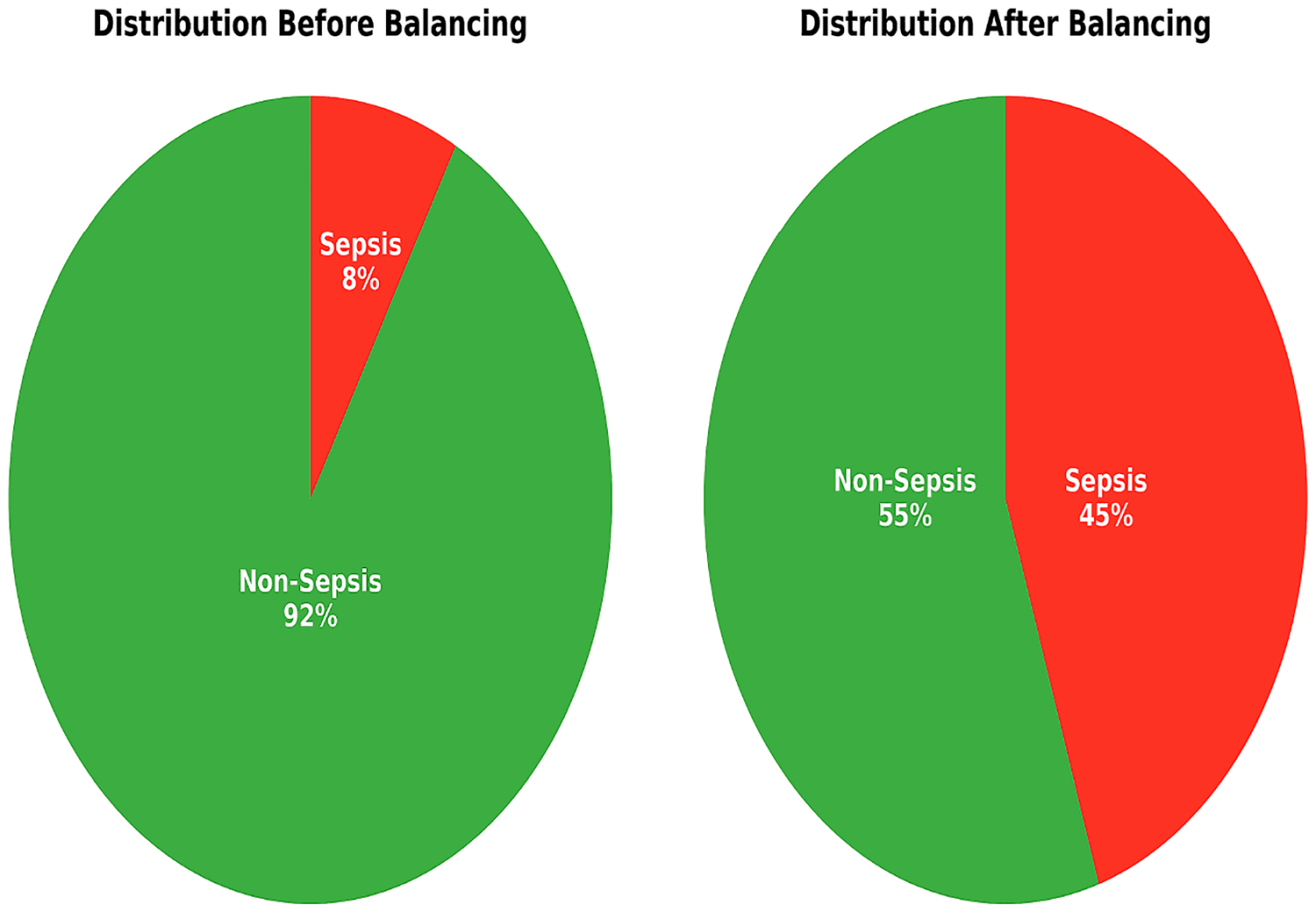
Class distribution before and after applying the two-stage balancing strategy.

This controlled approach to class balancing minimizes the risk of overfitting to the majority class while ensuring the model learns meaningful patterns from both sepsis and non-sepsis examples, as demonstrated in Fig. 6.

**Fig. 7:**
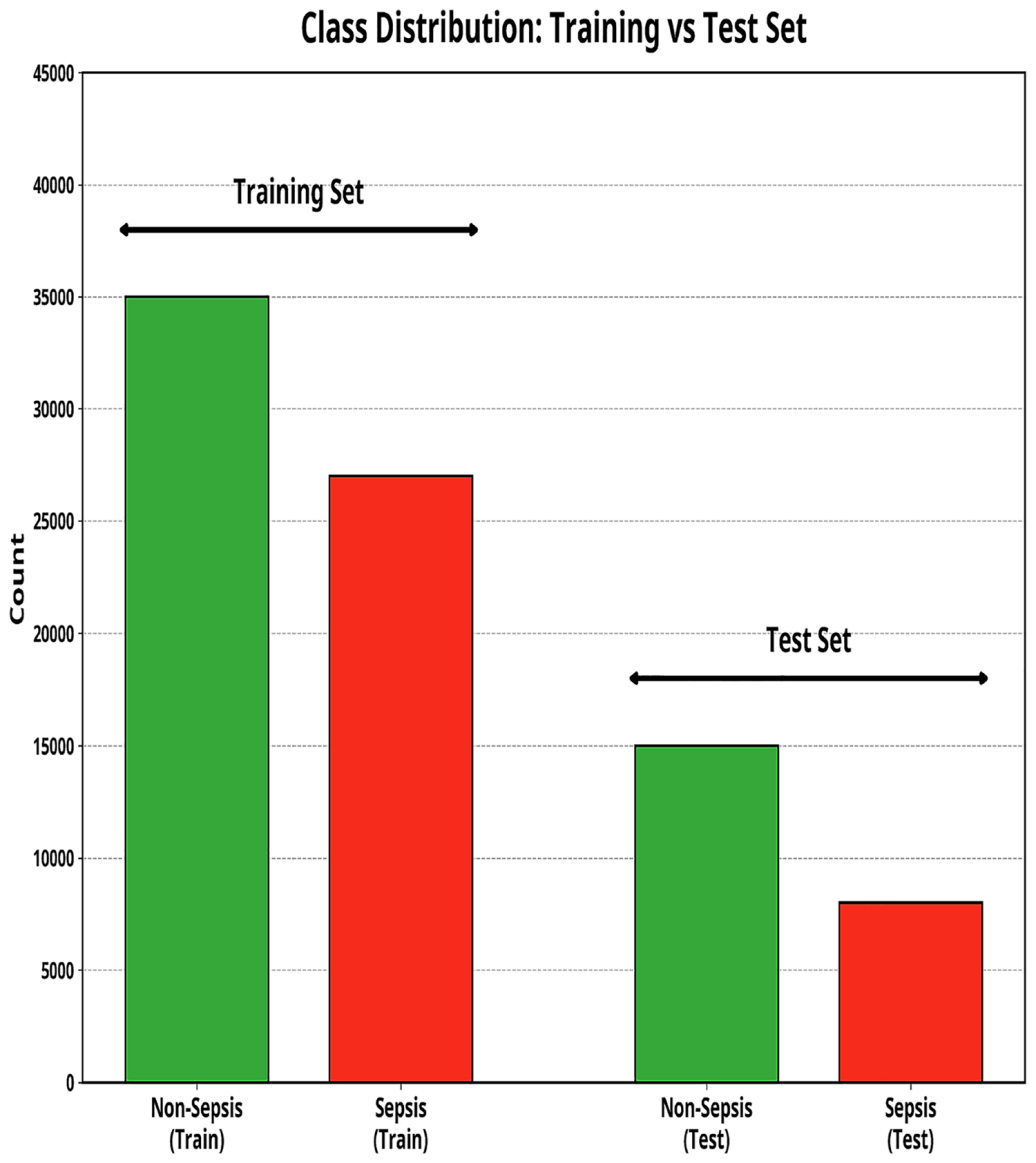
Class distribution after applying the balancing strategy.

The dataset was customized to control the number of samples from each class, reducing bias caused by class imbalance. This preprocessing step ensures more balanced training and testing sets, thereby promoting fairer model evaluation and improved generalization performance across both classes, as shown in Fig. 7.

Moreover, this class balancing strategy enhances the model’s sensitivity to minority class instances, which is essential for the timely and accurate detection of sepsis. It also enables a more reliable assessment of the model’s clinical effectiveness under realistic deployment scenarios, where early identification of rare cases can be critical.

This approach further minimizes the risk of overfitting to the majority class—a common challenge in medical datasets with a low prevalence of positive outcomes. By enforcing controlled class proportions, the model is better equipped to learn meaningful and generalizable patterns from both sepsis and non-sepsis examples.

**Fig. 8:**
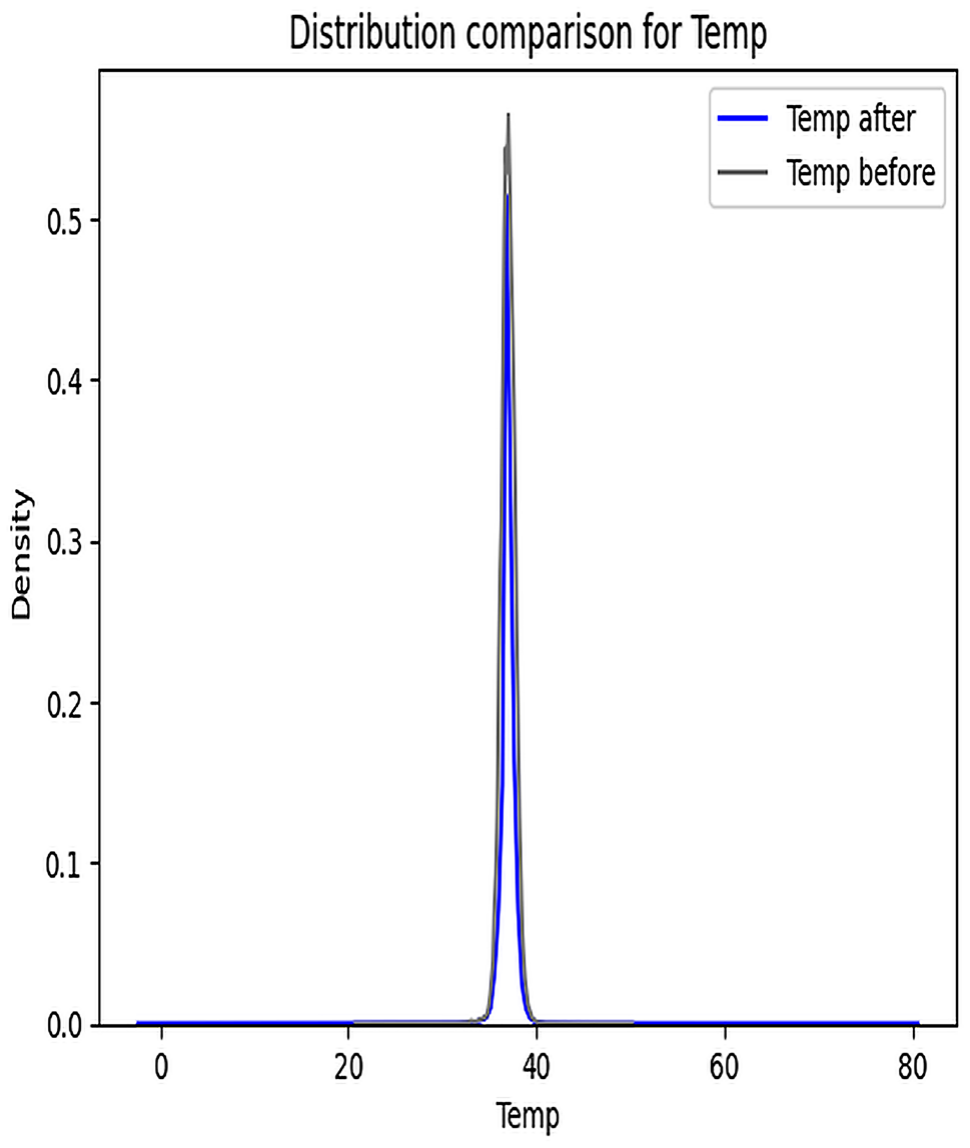
Histogram of temperature (Temp) feature distribution before (gray) and after (blue) preprocessing.

The distribution of the temperature (Temp) feature before and after preprocessing is shown in Fig. 8. The gray line represents the original distribution, while the blue line depicts the distribution after applying imputation and normalization. As seen, both lines follow a nearly identical trajectory, with peaks occurring at approximately the same value (37.5 °C). This overlap confirms that the preprocessing preserved the essential characteristics of the original temperature data without introducing significant distortion.

**Fig. 9:**
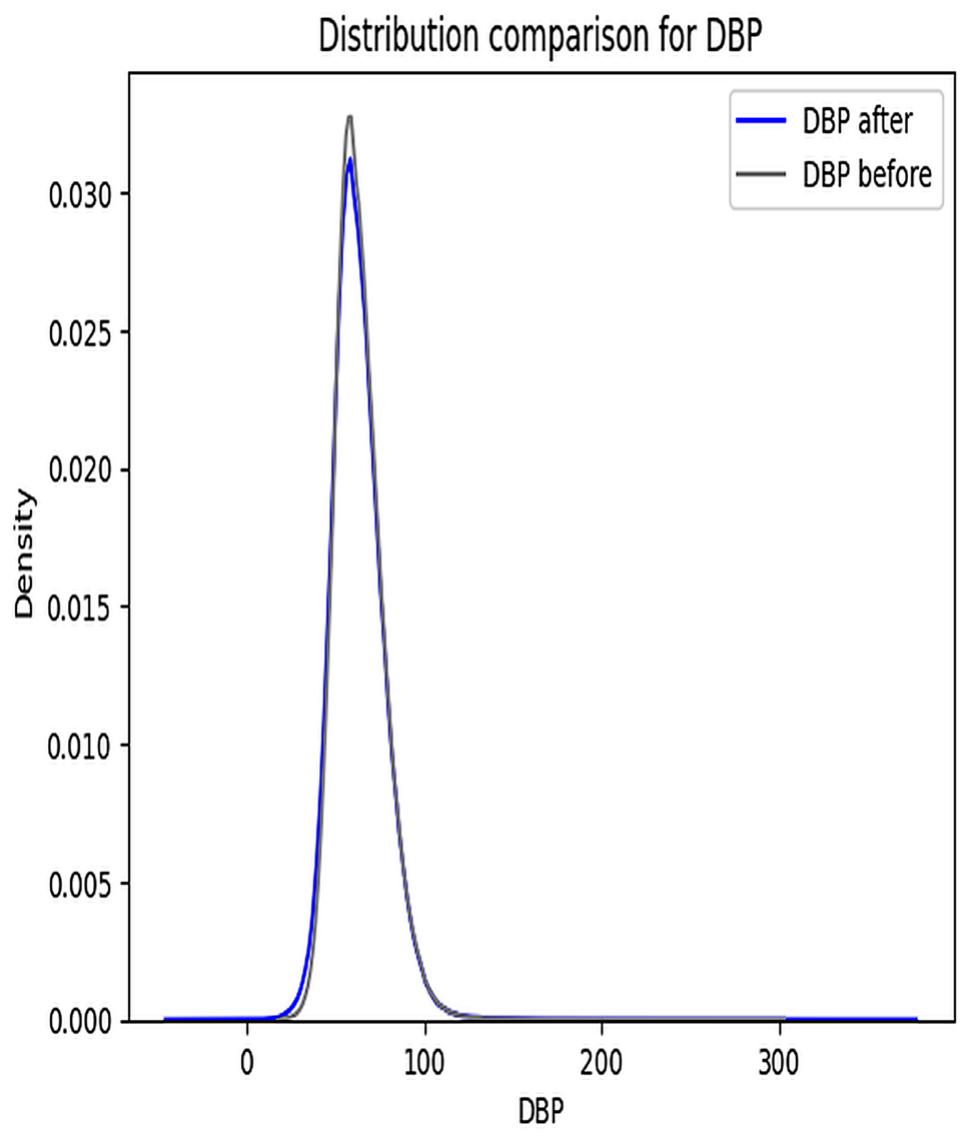
Histogram of diastolic blood pressure (DBP) feature distribution before (gray) and after (blue) preprocessing.

Similarly, Fig. 9 illustrates the distribution of the Diastolic Blood Pressure (DBP) feature before and after preprocessing. The alignment between the gray and blue curves is again prominent, particularly around the central region (75 mmHg). This consistency suggests that the imputation and scaling steps applied to the DBP feature successfully retained the original distribution’s structure, thus preserving the clinical integrity of the measurements.

**Fig. 10:**
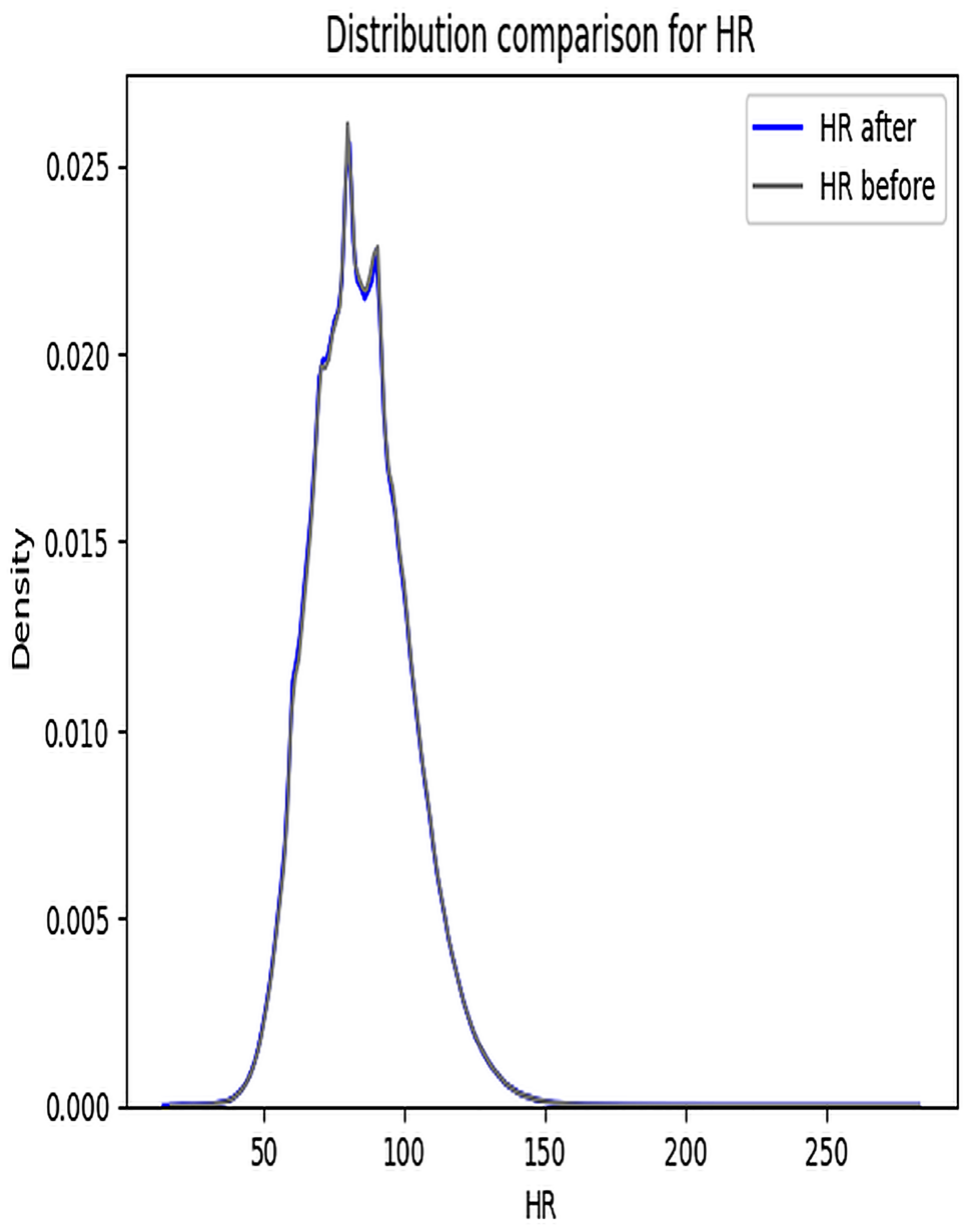
Distribution of heart rate (HR) feature before (gray) and after (blue) preprocessing.

In continuation, the Heart Rate (HR) feature displayed in Fig. 10 further confirms the stability of the preprocessing pipeline. Despite slight skewness in the heart rate values, the preprocessed (blue) and original (gray) curves demonstrate strong alignment across most of the range. This agreement reinforces the effectiveness of the preprocessing steps in preserving key physiological patterns across multiple vital signs.

Together, these results indicate that the preprocessing methodology maintained the essential distributional properties of critical features, ensuring data fidelity and minimizing bias introduced through imputation.

## Experimental results & discussion

### Experimental setup

All models were implemented in Python 3.9 and executed in a high-performance computing environment provided by Kaggle, leveraging GPU acceleration for efficient training and evaluation.

*Software and Frameworks* The experimental framework utilized Python 3.9 as the primary programming language with TensorFlow 2.13.0 (tensorflow.keras.layers) serving as the deep learning framework. The implementation incorporated NumPy for numerical computations, Pandas for data manipulation, Scikit-learn for machine learning utilities, Matplotlib for visualization, and SHAP and PFI for model interpretability analysis.

*Execution Environment* All training and evaluation procedures were conducted on the Kaggle Kernel (Free Tier) platform, equipped with an NVIDIA Tesla P100 GPU featuring 16 GB HBM2 memory. Hardware acceleration was enabled throughout the experimental process, with the runtime environment configured as a GPU-enabled notebook to ensure optimal computational performance.

*Development System* Initial model development and testing were performed on a local workstation prior to deployment on the Kaggle GPU environment. The local system specifications comprised an Intel Core i7-13650HX processor (13th generation, 14-core), NVIDIA GeForce RTX 3050 graphics card (6 GB GDDR6), 16 GB DDR5 RAM (2$\times$8 GB, 4800 MHz), 512 GB M.2 PCIe NVMe SSD storage, and 15.6-inch FHD display (1920$\times$1080) operating at 120 Hz refresh rate. This configuration provided an optimal balance between development flexibility and computational efficiency during data preprocessing and hyperparameter tuning phases.

*Reproducibility* Experimental reproducibility was ensured through systematic implementation of fixed random seeds using NumPy and TensorFlow random state initialization protocols. Model evaluation employed stratified 5-fold cross-validation methodology to guarantee robust and statistically reliable performance assessment across heterogeneous data partitions.

### Model performance results

Among time-series models, BiLSTM, TCN, and ResNet exhibit superior performance, as shown in Table 8 .

**Table 8:** Performance comparison of deep learning models for sepsis prediction (MIMIC-III Dataset).

Model/Variant	Balancing approach	AUROC	Recall (S)	NPV
TCN—Different balancing strategies				
TCN	Proposed balancing approach	0.9712	0.91	0.9865
TCN	Weighted Class	0.8219	0.44	0.9887
TCN	SMOTE	0.9887	0.85	0.9971
TCN	No sampling	0.7973	0.43	0.9883
BiLSTM—Different balancing strategies				
BiLSTM	Proposed balancing approach	0.9659	0.96	0.9943
BiLSTM	Weighted Class	0.9464	0.82	0.9963
BiLSTM	SMOTE	0.9744	0.82	0.9965
BiLSTM	No sampling	0.9402	0.83	0.9964
Baseline comparison (with proposed balancing approach)				
ResNet	Proposed balancing approach	0.9300	0.94	0.9906
LSTM	Proposed balancing approach	0.9574	0.95	0.9924
InceptionTime	Proposed balancing approach	0.9278	0.77	0.9666
1D CNN	Proposed balancing approach	0.9003	0.91	0.9843
GRU	Proposed balancing approach	0.8324	0.85	0.9718
RNN	Proposed balancing approach	0.8459	0.90	0.9807

(S), sepsis class; NPV, negative predictive value. All models evaluated on MIMIC-III Dataset.

To test and evaluate the models, 23,000 data points out of a total of 85,000 were kept hidden as an internal test set.

*BiLSTM* demonstrates strong performance with an F1 (S) score of 0.85, particularly in recall (0.95), indicating effective identification of positive instances (Table 8). Its precision (0.77) is comparable to that of TCN. The ROC-AUC score of 0.9566 further supports its robust discriminative ability. The model’s false negative (FN) count is 377 out of 23,000, highlighting its reliability in medical contexts where minimizing missed positive cases is critical^29^. The model benefits from bidirectional processing, which captures temporal context from both past and future data.

*TCN* achieves an identical F1 (S) score of 0.85, with precision of 0.77 and recall of 0.94 (Table 8). It records the highest ROC-AUC score of 0.9595 among all time-series models, reflecting excellent class separation. The FN count is slightly higher than BiLSTM at 480, yet still shows strong performance in minimizing undetected positives.

*ResNet* yields a lower F1 (S) score of 0.77 compared to TCN and BiLSTM, with precision at 0.75 and recall at 0.78 (Table 8). Its ROC-AUC is 0.8908, indicating relatively reduced discriminative capability. Moreover, the FN count stands at 1,350 out of 23,000, suggesting diminished effectiveness in capturing positive cases.

*Overall*, both TCN and BiLSTM stand out as the most effective time-series models. TCN offers a slight edge in ROC-AUC, whereas BiLSTM leads in recall, making it particularly suited for clinical tasks requiring high sensitivity (Table 8).

### XAI techniques

the top five models—TCN, BiLSTM, ResNet, LSTM, and InceptionTime—were selected for explainability analysis due to their strong overall performance in sepsis detection, as shown in Table 8.

#### TCN model explainability

As summarized in Table 8, TCN achieves an F1 (S) score of 0.85 and an F1 (NS) score of 0.90, with precision (S) of 0.77, recall (S) of 0.94, and ROC-AUC of 0.9595. It recorded 480 FNs, reflecting a strong ability to identify positive sepsis cases while performing well on negative cases.

**Fig. 11:**
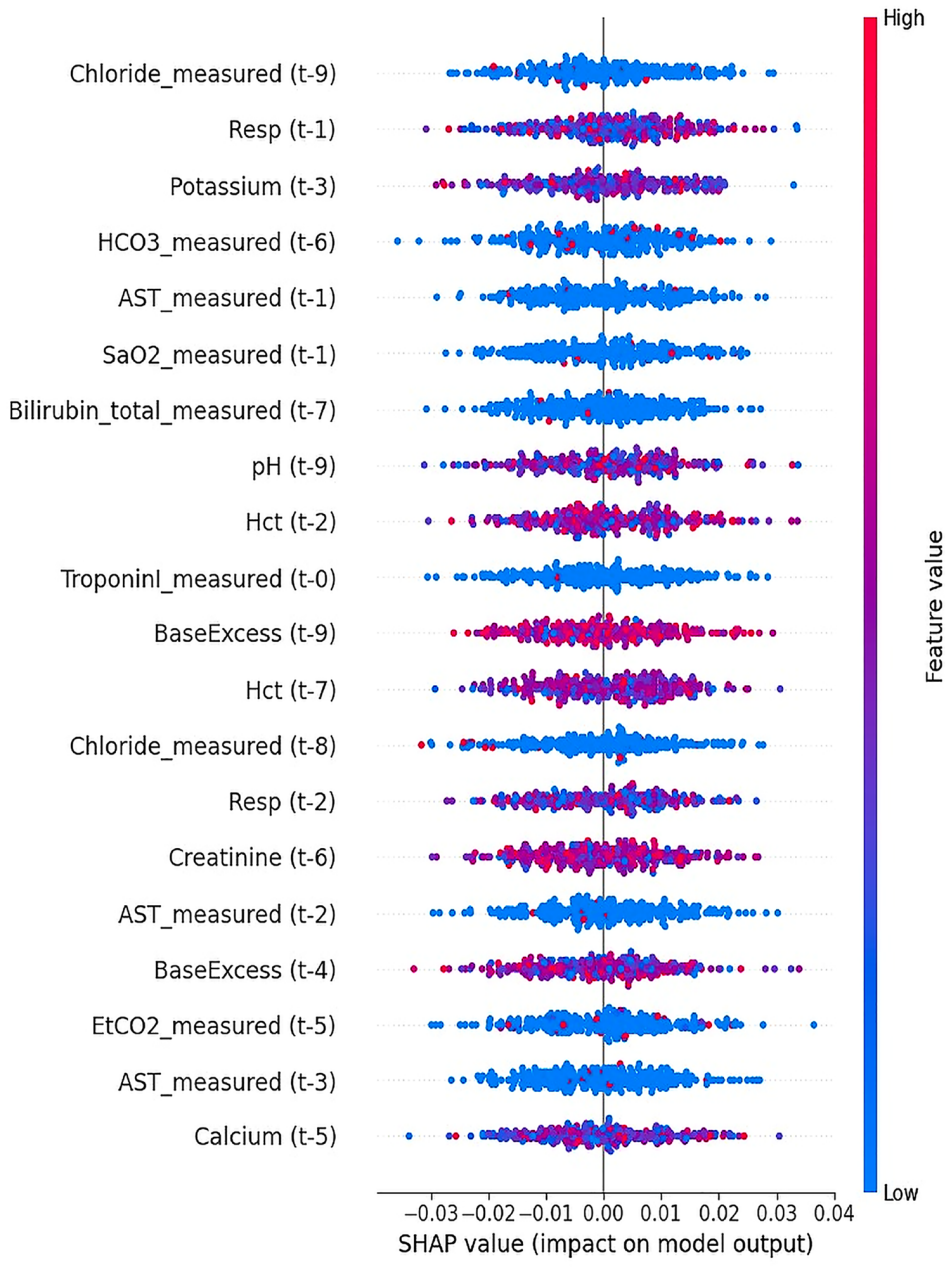
SHAP summary plot for TCN model showing clinical feature contributions to sepsis prediction.

SHAP employs game theory principles to fairly assign contribution values to input features for model outputs. For complex time-series models such as TCNs, SHAP quantifies the influence of each feature value at individual time steps on the final prediction, providing granular, instance-level explanations that are critical for clinical decision-making and real-time monitoring.

Figure 11 presents the SHAP summary plot for the TCN model. Each point corresponds to a prediction instance, with the x-axis indicating contribution magnitude and direction. Features are ranked from top to bottom by their importance. Colors represent the actual feature values (red: high, blue: low), and the feature names are tagged with their corresponding time step relative to the prediction time (e.g., t-1, t-9).

Figure 11 confirms that specific clinical measurements at certain time steps have a strong influence on the model’s sepsis prediction. Notably, **Chloride measured at time step t-9**, **Respiration at t-1**, **Potassium at t-3**, and **HCO**_**3**_ **measured at t-6** are among the key contributors. High Chloride (t-9) and high Respiration rate (t-1) are associated with an increased predicted risk of sepsis, while high Potassium levels (t-3) tend to decrease the risk.

These findings align well with established clinical knowledge: elevated respiration rate is a hallmark of systemic inflammatory response, and abnormal chloride levels are often seen in patients with metabolic imbalance linked to sepsis. Potassium levels, while variable, may reflect different physiological states, with lower values often linked to critical illness. Therefore, the SHAP plot not only explains the model’s decision process but also validates it against several medically known sepsis indicators and risk patterns.

**Fig. 12:**
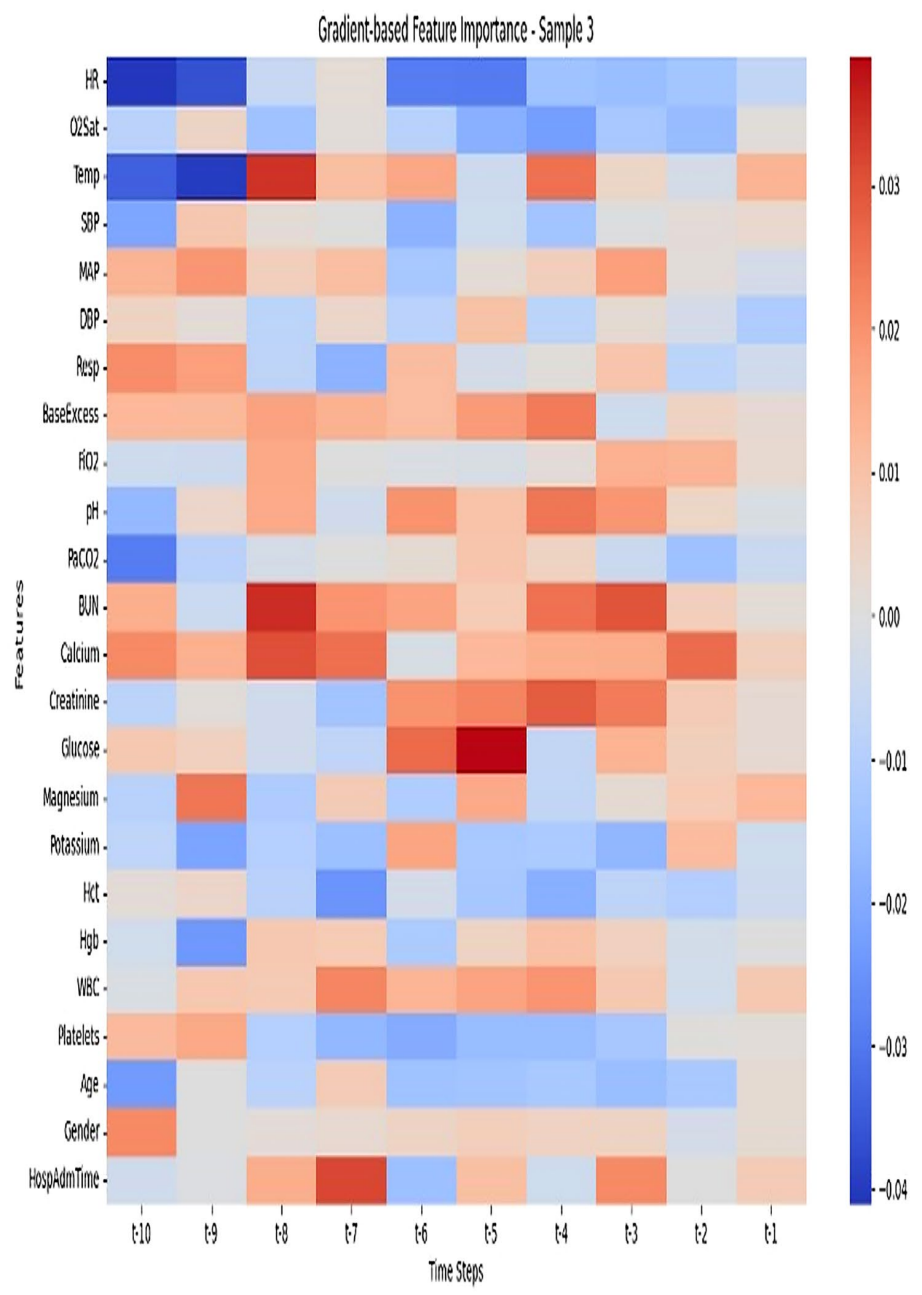
Gradient-based feature-importance heatmap for a representative test-set sample using the trained TCN model. The heatmap was generated by computing the gradient of the model’s predicted sepsis probability with respect to each input feature at each time step using TensorFlow GradientTape v2.13.0 (https://www.tensorflow.org/api_docs/python/tf/GradientTape).

Gradient-based methods compute the sensitivity of model output to input feature perturbations, revealing important time windows and key features influencing predictions for temporal models such as TCNs.

Figure 12 shows a gradient-based heatmap illustrating feature sensitivity across time steps for a test sample. Warmer colors indicate positive influence, cooler colors indicate negative influence, and paler areas indicate low sensitivity. Vital signs, including temperature, O_2_Sat, HR, SBP, DBP, and respiration, show high sensitivity at times t-8, t-5, and t-2. Laboratory values such as BUN, creatinine, potassium, and hematocrit are influential around t-8 and t-3. This visualization highlights the temporal dynamics of clinical features affecting sepsis risk prediction.

#### BiLSTM model explainability

As summarized in Table 8, BiLSTM achieves an F1 (S) score of 0.85, a recall of 0.95, a precision of 0.77, and ROC-AUC of 0.955, demonstrating strong positive case identification capacity.

**Fig. 13:**
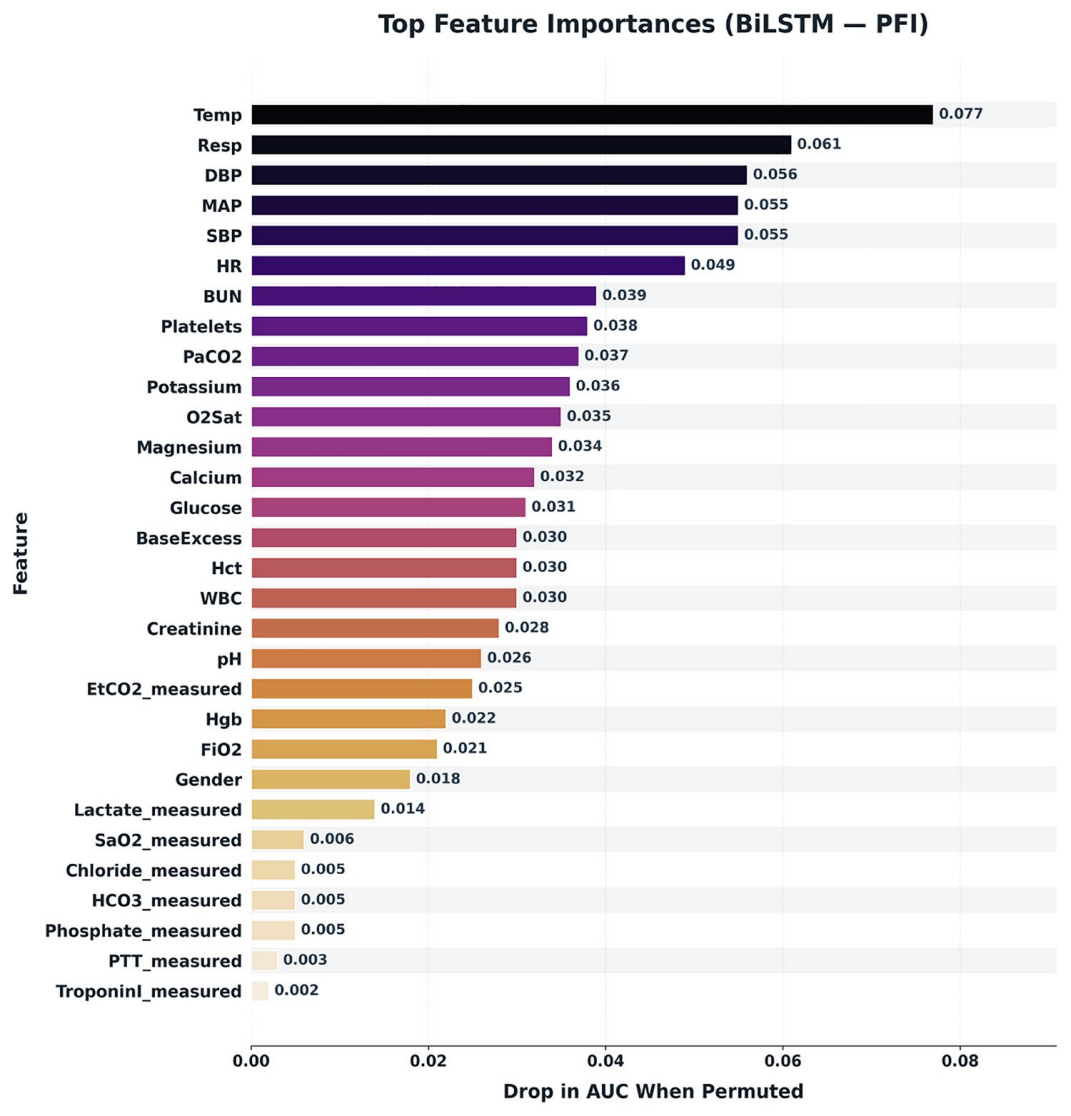
PFI analysis for BiLSTM model showing clinical feature contributions to sepsis prediction.

SHAP was not applied to the BiLSTM model due to its complex sequential memory structure, which makes instance-level attribution less interpretable in a global context. Instead, PFI was selected to capture the aggregate influence of each feature on model predictions. PFI analysis in Fig. 13 ranks clinical features by their impact on BiLSTM predictions, with temperature as most important, followed by mean arterial pressure, respiration rate, and SBP.

#### ResNet model explainability

As summarized in Table 8, ResNet attains an F1 (S) score of 0.77, precision of 0.75, recall of 0.78, and ROC-AUC of 0.8908, reflecting moderate performance in positive sepsis identification.

**Fig. 14:**
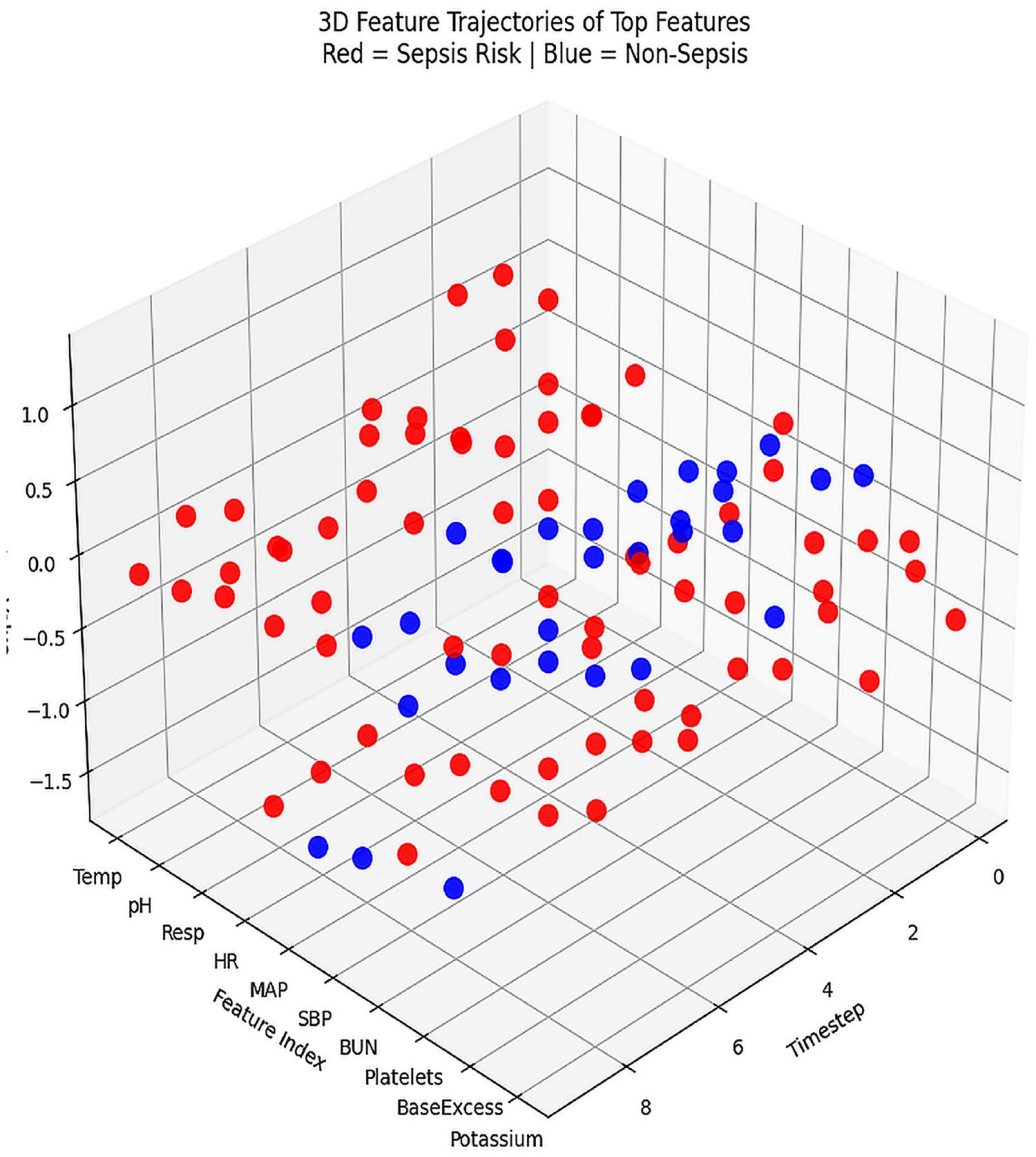
3D temporal feature trajectories in sepsis prediction (Red: Sepsis Cases, Blue: Non-Sepsis Controls).

Figure 14 illustrates a three-dimensional visualization of temporal evolution of clinical features used in sepsis prediction. The x-axis represents time-step progression (0-8), the y-axis shows various features including temperature, respiratory rate, MAP, HR, pH, DBP, hematocrit, hemoglobin, platelets, and SBP. The z-axis corresponds to normalized feature importance values (− 1.5 to + 1.5). Red and blue points denote sepsis cases and non-sepsis controls, respectively. This visualization reveals distinct temporal patterns and feature trajectories that differentiate clinical outcomes, emphasizing the dynamic nature of sepsis progression and time-dependent relationships among variables. By capturing how the predictive value of biomarkers evolves, this 3D representation supports identification of critical feature combinations and lead-lag relationships, enhancing predictive modeling and early warning systems.

#### LSTM model explainability

As shown in Table 8, the LSTM model achieves an F1 (S) score of 0.80, with a precision of 0.78, a recall of 0.82, and an ROC-AUC of 0.8957, indicating solid performance in identifying positive sepsis cases.

**Fig. 15:**
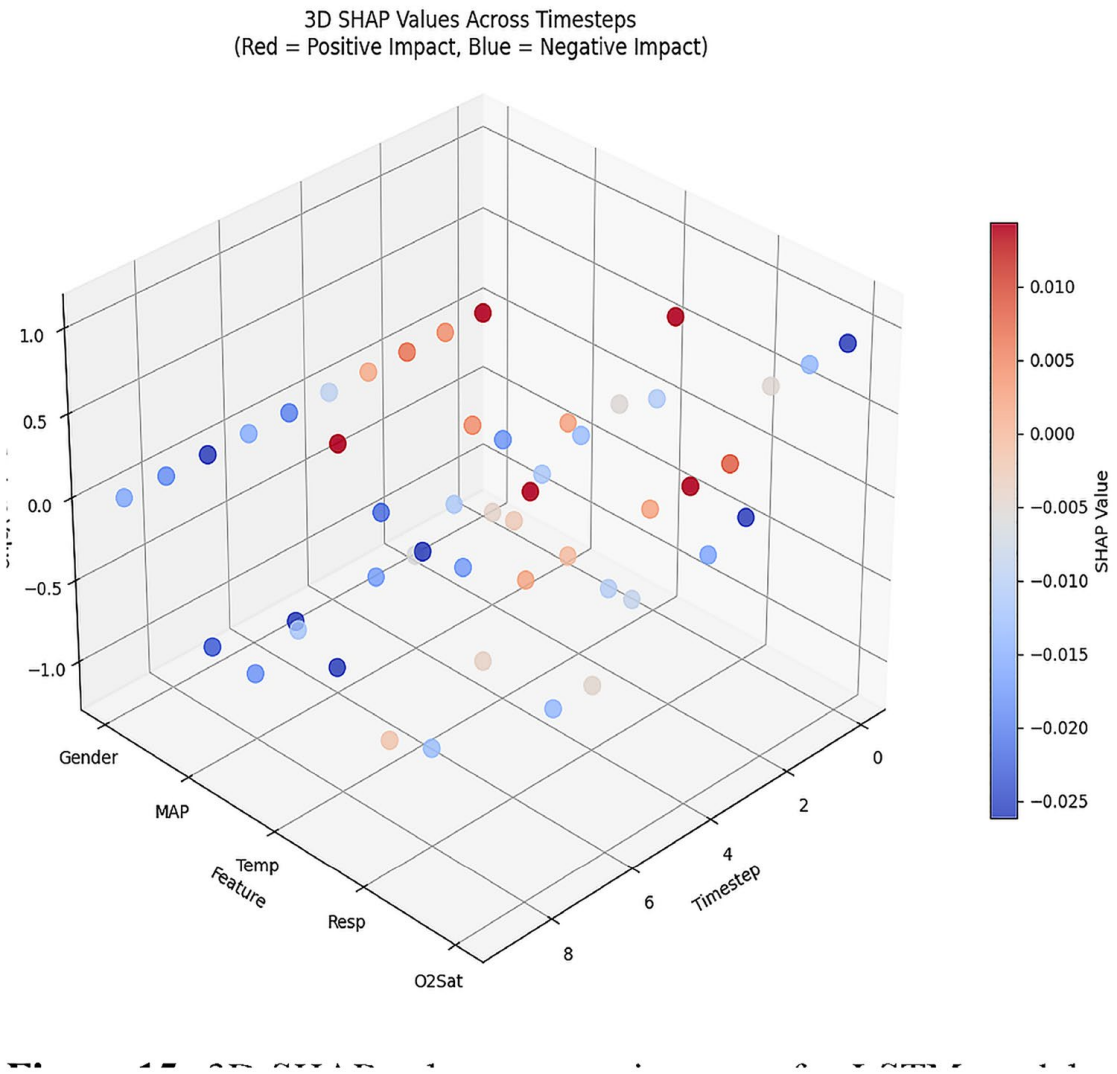
3D SHAP values across timesteps for LSTM model (Red: Positive Impact, Blue: Negative Impact).

Figure 15 visualizes the temporal evolution of feature importance across multiple timesteps (0–8) for the LSTM model using 3D SHAP analysis. This visualization illustrates how critical features—HR among them—contribute to the model’s prediction. O_2_Sat, Temperature, Respiratory Rate (Resp), and Platelets contribute to sepsis prediction over time. Red points indicate positive SHAP values (features pushing toward sepsis prediction), while blue points represent negative SHAP values (features pushing the prediction away from sepsis).

The color intensity, ranging from − 0.025 to + 0.015, reflects the magnitude of each feature’s contribution. This temporal perspective reveals how the predictive importance of vital signs evolves throughout the monitoring period, enabling clinicians to understand which biomarkers become more critical at different stages of patient observation and how the LSTM model weighs these dynamic relationships for early sepsis detection.

**Fig. 16:**
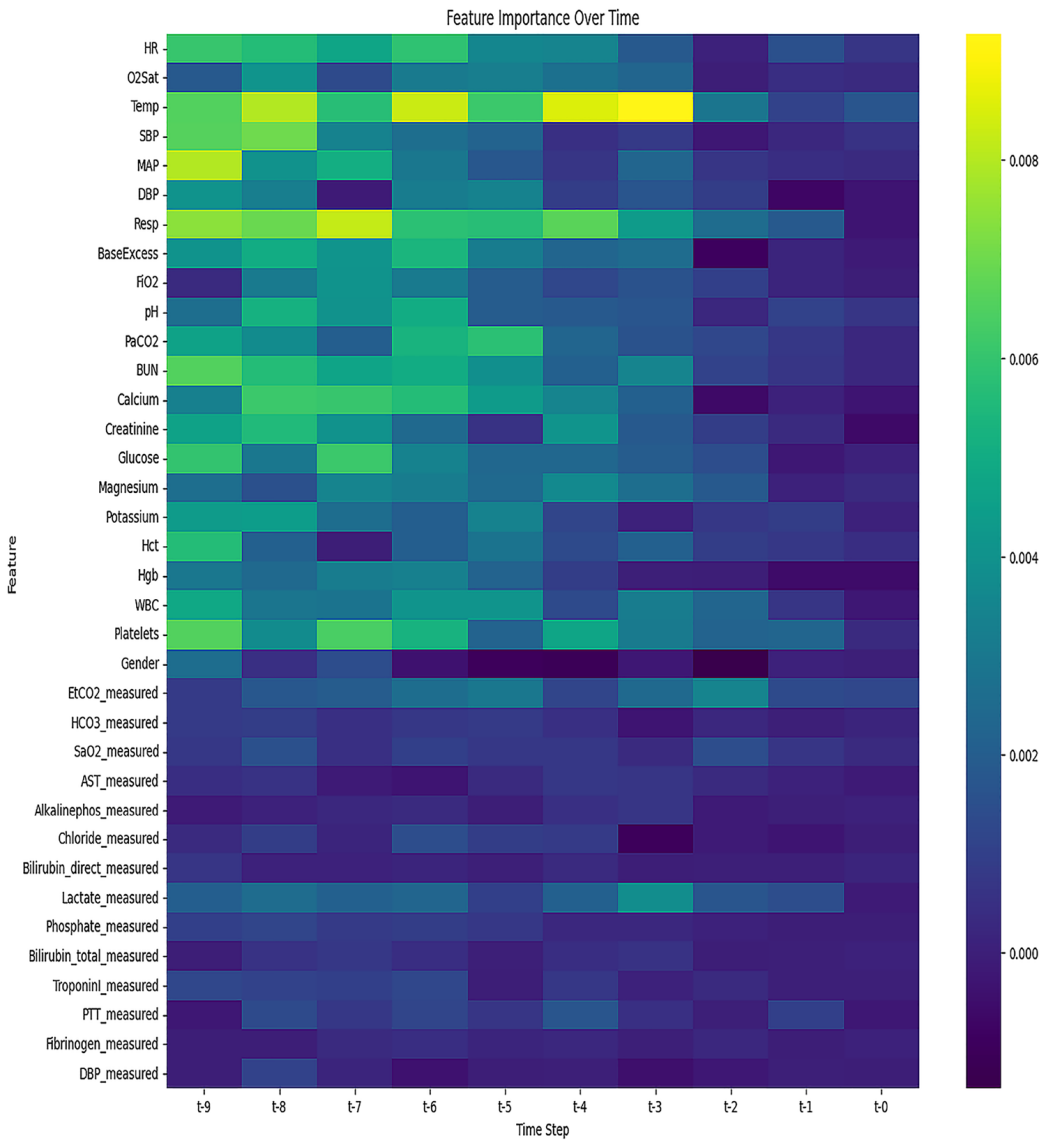
SHAP-based feature-importance heatmap for the test-set samples using the trained LSTM model. The heatmap was generated by computing the mean absolute SHAP values of the model’s predicted sepsis probability with respect to each input feature at each time step using the SHAP library v0.41.0 (https://shap.readthedocs.io/).

The heatmap in Fig. 16 displays the temporal evolution of feature importance across timesteps (t-9 to t-0) for LSTM model in sepsis prediction. This visualization reveals how predictive significance of clinical features changes over time, with importance values ranging from 0.000 to 0.010 shown in a color gradient from dark blue to bright yellow. HR, Temperature, and Respiratory Rate (Resp) emerge as critical features, demonstrating high importance in early time steps, while O_2_Sat and blood pressure parameters maintain consistent relevance throughout the monitoring period. Temporal patterns reveal that vital signs demonstrate peak importance during middle timesteps (t-7 to t-5). Laboratory parameters, particularly electrolytes and biomarkers, demonstrate diverse temporal importance patterns. Capturing these dynamics is critical for optimizing the performance of LSTM model as it helps identify which clinical parameters become most predictive at different stages of patient monitoring and supports development of time-sensitive early warning systems for sepsis detection.

#### InceptionTime model explainability

As shown in Table 8,The InceptionTime model achieves an F1 (S) score of 0.76, with a recall of 0.86, a precision of 0.68, and an ROC-AUC of 0.9014 on a test set of 23,000 samples.

**Fig. 17:**
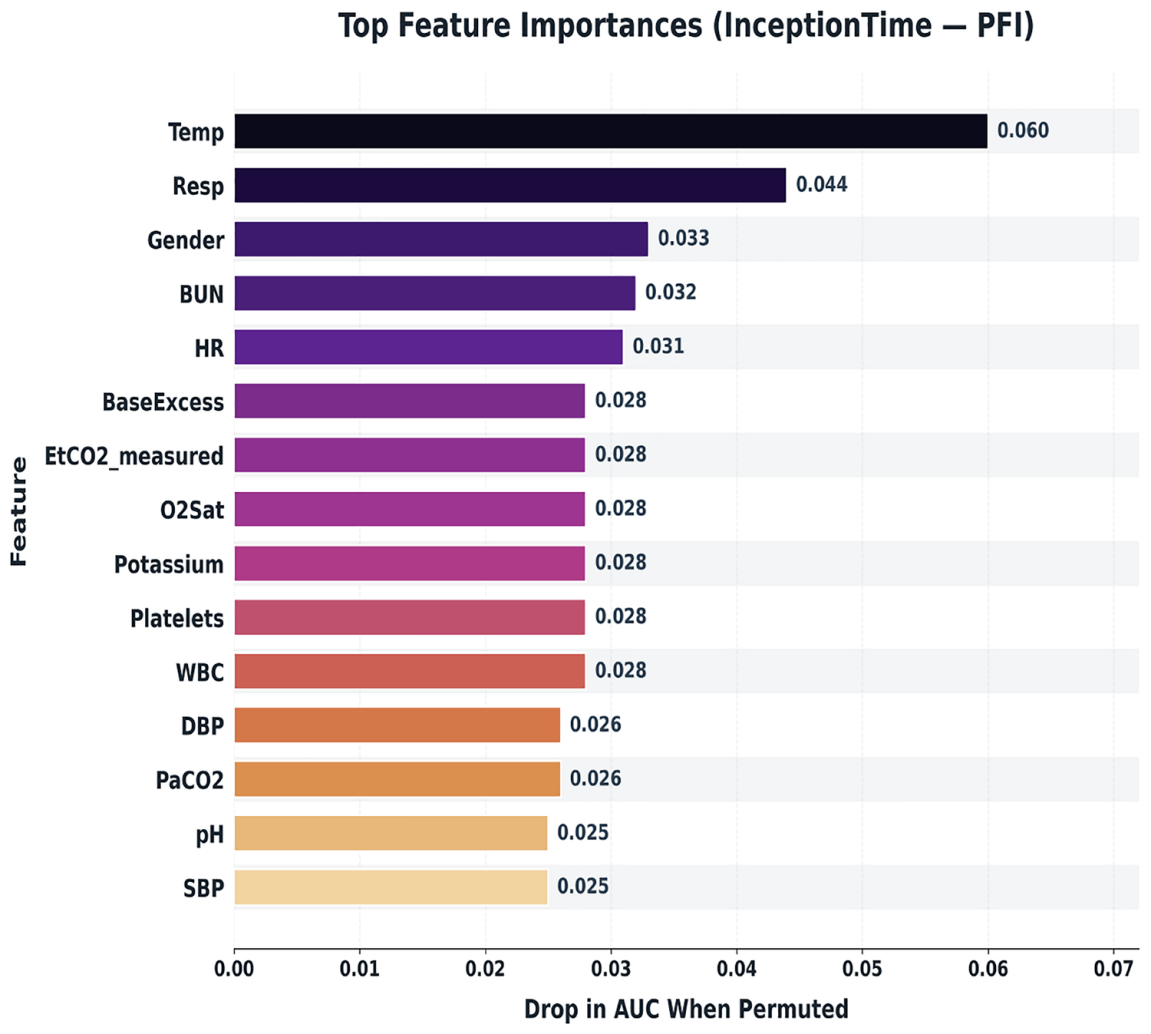
PFI analysis for InceptionTime model showing clinical feature contributions to sepsis prediction.

PFI analysis in Fig. 17 displays the contribution of clinical features to the model’s sepsis prediction performance, with temperature as the most critical feature, followed by respiration rate and gender.

### Discussion

Recent studies demonstrate that innovative approaches can be found for early diagnosis of sepsis by applying advanced computer algorithms^3,10,14^. Research indicates that intelligent systems are more adept at evaluating medical data across complex timescales^12,14,22^. Advanced neural network models have shown promising results in this regard. BiLSTM and TCN models have achieved remarkable results, with F1 ratios reaching approximately 0.85, surpassing traditional methods such as logistic regression (0.65) and RF (0.69)^8,14^, also, overperforming the reported results in the literature as shown in Table 9.

What distinguishes these intelligent systems is their exceptional ability to accurately detect true cases, with recall ratios ranging between 0.94 and 0.95, reflecting high efficiency in detecting truly infected patients^18^. This superiority is due to fundamental technical advances in computer architecture, as these systems are capable of understanding complex temporal patterns present in electronic patient records. Bidirectional model analyzes preceding and subsequent temporal indicators, while temporal analysis networks rely on advanced algorithms to extract temporal patterns efficiently without need for expensive and complex computational operations.

Preliminary data processing methods played a key role in significantly improving performance. Researchers used advanced techniques to balance samples and generate synthetic data that mimics real biomarkers^6,7^, as well as intelligent strategies to address missing data while preserving original clinical context^16^. Unlike traditional methods that ignore missing data, the research team considered these gaps to be clinically meaningful indicators that reflect real-world diagnostic decisions made by clinicians in practice^12^.

New methods used in this research demonstrate significant progress compared to previous work, achieving a 13% increase in accuracy compared to studies that relied on gradient boosting algorithms^19,21^.

This work contributes to development of an integrated diagnostic system that combines high scientific accuracy with easy interpretation of results.

New analytical approach, powered by multiple XAI techniques, effectively revealed complex and time-dependent relationships between a variety of diagnostic indicators. For instance, SHAP analysis revealed significant temporal influence of key vital signs such as Temp, HR, SBP, MAP, and DBP at various time steps leading up to prediction (Fig. 11). Similarly, gradient-based heatmaps identified critical time windows where model’s sensitivity to fluctuations in these same vital signs peaked, which helped distinguish sepsis from non-sepsis trajectories (Fig. 12). These granular, time-aware insights consistently ranked vital signs—Temperature (Temp), HR, SBP, MAP, and DBP—as top global predictors across models (Figs. 13, 17), enabling design of more intelligent medical monitoring systems responsive to dynamic and subtle evolution of patient’s condition.

The effectiveness of these vital signs in sepsis detection is well established in the literature. Temperature alterations, both hyperthermia (> 38 °C) and hypothermia (< 36 °C), are fundamental sepsis criteria with odds ratios (OR) of 2.126 for elevated temperature^30,31^. HRV analysis has demonstrated remarkable predictive capabilities, with studies showing that HR combined with temperature achieves area under the curve values of 0.94 for sepsis prediction^32^. Additionally, heart rate characteristics monitoring has proven effective for early detection, particularly in neonatal sepsis, where decreased HRV punctuated by transient decelerations serves as a reliable biomarker^33,34^.

Blood pressure components provide crucial hemodynamic insights for sepsis assessment. SBP maintained at approximately 140 mmHg during the first 10 hours of hospitalization has been associated with decreased mortality risk^35,36^. MAP serves as a critical perfusion indicator, with studies demonstrating that MAP below 70 mmHg carries an OR of 3.874 for sepsis development^30^. DBP has emerged as particularly significant, with values below 59 mmHg associated with increased 28-day mortality (OR 1.915) and serving as a marker of arterial tone and coronary perfusion adequacy^37,38^.

A combination of these vital signs provides synergistic diagnostic value. Research has shown that a minimal feature set yielding maximal predictability combines HR and temperature, achieving sensitivity of 0.85 and specificity of 0.90^32^. Furthermore, shock index (HR/SBP) significantly correlates with sepsis severity, with increased values strongly influencing Sequential Organ Failure Assessment (SOFA) scores^30,39^.

**Table 9:** Literature comparison of deep learning models for sepsis prediction.

Model	Study	AUROC	Recall (S)	NPV
MGP-TCN	Lee et al.^26^	0.915	–	–
TCN	Apalak & Kiasaleh^27^	0.924	–	–
BiLSTM	Venkatesh et al.^28^	–	0.95	–

(S), sepsis class; NPV, negative predictive value. ‘–’ indicates metric not reported.

#### Limitations

Despite these promising developments, the study faces several fundamental challenges. A key limitation of this study is the absence of external validation on independent clinical cohorts. The proposed models were developed and evaluated exclusively using the PhysioNet 2019 Challenge dataset, which, although large and widely used, represents a specific aggregation of ICU populations, clinical practices, and data acquisition protocols. Differences in patient demographics, monitoring frequency, institutional treatment practices, and data recording standards across healthcare systems may affect model generalizability. Due to the lack of publicly available ICU time-series datasets with comparable variable definitions, temporal resolution, and sepsis labeling criteria, direct external validation was not feasible within the scope of this study. As a result, the findings should be interpreted as a methodological and research validation rather than evidence of immediate clinical deployment readiness.

Synthetic data augmentation introduces potential risks of bias and artificial pattern reinforcement if not carefully controlled. Perturbation-based augmentation may amplify existing correlations or introduce unrealistic feature combinations. To mitigate this risk, augmentation was restricted to features identified as clinically informative through feature importance estimation, and perturbations were bounded to physiologically plausible ranges.

Furthermore, a lack of diversity in the original training samples may affect the generalizability of results to other medical conditions. This shortcoming highlights the urgent need for in-depth research to expand the database and improve the efficiency of the proposed diagnostic system. A deeper clinical evaluation of the interpretation mechanisms used in the study is also required. Next steps should focus on application aspects, including integrating these systems with existing medical software and developing interactive interfaces that meet requirements for use in clinical settings.

Practically, this strategy represents an important advance in the development of effective early warning systems in intensive care units, which may contribute to reducing mortality rates resulting from sepsis^1,4^. Furthermore, this strategy presents an innovative model for applying AI technologies in healthcare by balancing scientific rigor with practical relevance, a critical factor for successful application of these technologies in intensive care settings.

## Conclusion & future work

This work offers a comprehensive system for predicting sepsis in its early stages by integrating interpretable artificial intelligence (AI) methods alongside sophisticated machine learning (ML) algorithms. By improving system performance for solving common problems in clinical datasets, such as missing data and imbalanced classes^3,25^, we developed preprocessing workflows tailored to real-world clinical irregularities. Temporal deep learning models BiLSTM and TCN perform best with F1 scores of 0.84 and 0.85, and ROC-AUC scores of 0.955 and 0.966, respectively (Table 8). These models bolster performance in capturing the dynamic nature of physiological signals, critically reducing false negatives in life-threatening situations where early detection is paramount^1,4^. The temporal modeling capability of these architectures enables identification of subtle time-based patterns that traditional methods often miss^8,14^, providing clinicians with more reliable early warning systems for sepsis onset. To enhance interpretability and clinician trust, a variety of interpretable AI methods, including SHAP, PFI, and other gradient and cumulative local effect methods were used to reveal not only which features were most predictive but also when they became most critical in a patient’s timeline, as shown in our XAI analyses (Figs. 11–17).

Looking ahead, testing Selected Models within real intensive care unit (ICU) environments will be a crucial step in confirming their validity and robustness across a wide range of clinical scenarios. Predictive models could also be incorporated into clinical workflows through intuitive dashboards that provide explanations and forecasts, thus improving decision-making at the bedside. Including longitudinal patient records as well as comprehensive medical histories could improve the accuracy of predictions and generalizability. Other temporal models could also enhance these improvements using ensemble learning strategies that draw on multiple temporal models. The ability to provide proactive interventions could be possible with real-time adaptive monitoring systems that continuously revise predictions based on new patient information^12^. Addressing ethical issues like algorithmic bias and patient privacy will need to be attended to in order for AI technologies to be responsibly utilized in the healthcare sector^40,41^. With these considerations, the goal is to develop reliable, transparent, and clinically actionable AI systems for early sepsis detection that can meaningfully improve patient outcomes in critical care settings^22,42^.

## Data Availability

The training dataset used in this study, consisting of 1.5 million ICU observations from over 40,000 patients, has been merged from two sources. The merged dataset is publicly available through PhysioNet and can be accessed at the following URL: https://physionet.org/content/challenge-2019/1.0.0/.

## Code availability

The code used for data preprocessing, model implementation, and analysis in this study will be made available upon reasonable request to the corresponding author.
